# Automatic Human Sleep Stage Scoring Using Deep Neural Networks

**DOI:** 10.3389/fnins.2018.00781

**Published:** 2018-11-06

**Authors:** Alexander Malafeev, Dmitry Laptev, Stefan Bauer, Ximena Omlin, Aleksandra Wierzbicka, Adam Wichniak, Wojciech Jernajczyk, Robert Riener, Joachim Buhmann, Peter Achermann

**Affiliations:** ^1^Chronobiology and Sleep Research, Institute of Pharmacology and Toxicology, University of Zurich, Zurich, Switzerland; ^2^Neuroscience Center Zurich, University of Zurich and ETH Zurich, Zurich, Switzerland; ^3^Center for Interdisciplinary Sleep Research, University of Zurich, Zurich, Switzerland; ^4^Information Science and Engineering, Institute for Machine Learning, ETH Zurich, Zurich, Switzerland; ^5^Max Planck Institute for Intelligent Systems, Tübingen, Germany; ^6^Sensory-Motor Systems Lab, ETH Zurich, Zurich, Switzerland; ^7^Sleep Disorders Center, Department of Clinical Neurophysiology, Institute of Psychiatry and Neurology in Warsaw, Warsaw, Poland; ^8^Third Department of Psychiatry and Sleep Disorders Center, Institute of Psychiatry and Neurology in Warsaw, Warsaw, Poland; ^9^University Hospital Balgrist (SCI Center), Medical Faculty, University of Zurich, Zurich, Switzerland

**Keywords:** deep learning, sleep, EEG, automatic scoring, random forest, artificial neural networks, features, raw data

## Abstract

The classification of sleep stages is the first and an important step in the quantitative analysis of polysomnographic recordings. Sleep stage scoring relies heavily on visual pattern recognition by a human expert and is time consuming and subjective. Thus, there is a need for automatic classification. In this work we developed machine learning algorithms for sleep classification: random forest (RF) classification based on features and artificial neural networks (ANNs) working both with features and raw data. We tested our methods in healthy subjects and in patients. Most algorithms yielded good results comparable to human interrater agreement. Our study revealed that deep neural networks (DNNs) working with raw data performed better than feature-based methods. We also demonstrated that taking the local temporal structure of sleep into account a priori is important. Our results demonstrate the utility of neural network architectures for the classification of sleep.

## Introduction

### Problem Statement

Visual scoring of the sleep stages is the gold standard in sleep research and medicine. Sleep scoring is performed visually based on the following signals: (1) electrical activity of the brain – electroencephalogram (EEG), (2) electrical activity resulting from the movement of the eyes and eyelids – electrooculogram (EOG) and (3) muscle tone recorded under the chin (submental) – electromyogram (EMG).

Sleep scoring is usually performed according to standardized scoring rules: Rechtschaffen and Kales (1968) or the American Association of Sleep Medicine (AASM) (Iber et al., 2007). According to the AASM rules (Iber et al., 2007) an expert visually classifies consecutive 30-s epochs of polysomnographic (PSG) data (EEG, EOG and EMG) into wake, rapid eye movement (REM) sleep, and non-REM (NREM) sleep (stages N1–N3). If scoring is performed according to Rechtschaffen and Kales (1968), 20- or 30-s epochs are scored and NREM sleep is subdivided into stages 1–4 with stages 3–4 considered as slow wave sleep (SWS, deep sleep, corresponding to N3). Furthermore, Rechtschaffen and Kales (1968) defined movement time as a separate stage.

**FIGURE 1:**
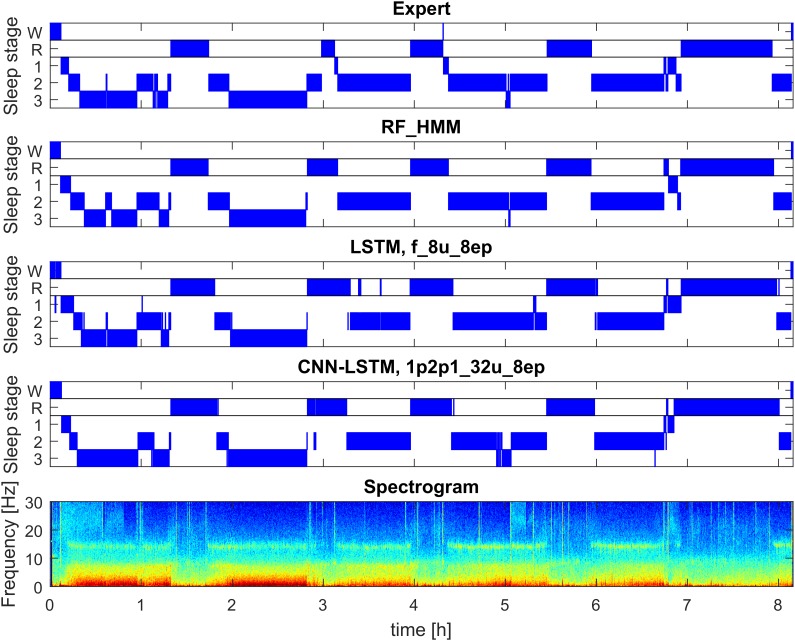
Example of automatic sleep scoring trained on healthy subjects (dataset 1; example from validation set). Panel 1: hypnogram (W, waking; R, REM sleep, 1–3: NREM sleep stages N1–N3) scored by a human expert. Panel 2: hypnogram resulting from RF classification based on features followed by temporal smoothing with HMM. Panel 3: hypnogram resulting from classification with 3-layer bidirectional LSTM network with 8 LSTM neurons in each layer based on features, sequence length eight epochs (i.e., 160 s). Panel 4: hypnogram resulting from a CNN-LSTM network with 11 convolutional layers and 2-layer bidirectional LSTM with 32 LSTM neurons in each layer. Input comprised of raw data (1 EEG and 2 EOG) and EMG power (1 value per epoch). Bottom panel: spectrogram (power density spectra of 20-s epochs color-coded on a logarithmic scale [0 dB = 1 μV^2^/Hz; –10 dB 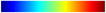 20 dB]) of EEG derivation C3A2. See Supplementary Material for the naming conventions of the algorithms.

The plot of a sequence of sleep stages is called a hypnogram (see Figure 1). Human sleep starts generally with a stage 1 (N1), which usually lasts only up to a few min and is a very light sleep. Slow rolling eye movements are a feature of stage 1 and contractions of the muscles, hypnagogic jerks may occur.

Next follows stage 2 (N2), a deeper state of sleep than stage 1, characterized by the occurrence of sleep spindles and K-complexes and an intermediate muscle tone.

Stage 2 usually precedes deep sleep – stages 3 and 4 (SWS, N3). The main characteristic of deep sleep is the presence of slow oscillations (<1 Hz) and delta waves (1–4 Hz) in the EEG for at least 20% of the epoch duration. The muscle tone is low.

Rapid eye movement sleep occurs periodically throughout the night and is characterized by rapid eye movements, fast low-amplitude EEG activity like the wake EEG, and a low muscle tone (atonia).

The progression of the different stages is not random, but rather follows a cyclic alternation of NREM and REM sleep (Achermann and Tarokh, 2014) with a cycle duration of approximately 90 min (see Figure 1 for a typical structure). Healthy sleep consists of approximately 3–5 sleep cycles.

Visual scoring by an expert is time consuming and subjective. Several studies addressed the interrater reliability and revealed that correspondence between scorers is far from ideal (Danker-Hopfe et al., 2004; Penzel et al., 2013; Rosenberg and Van Hout, 2013; Younes et al., 2016, 2018).

Several measures can be used to compare two experts or an algorithm with an expert. The simplest one is accuracy, the proportion of epochs which were assigned the same sleep stage. The F1 score (Dice, 1945; Sørensen, 1948) is a measure computed per class and it is widely used in the field of machine learning, and was also applied to assess performance in automatic sleep scoring (Tsinalis et al., 2016; Supratak et al., 2017; Chambon et al., 2018).

It was argued that F1 score has certain disadvantages by Powers (2014). Cohen’s kappa (Cohen, 1960) is a metric accounting for the agreement by chance and thus for imbalanced proportions of different classes and is commonly used in biology and in sleep research. Values higher than 0.8 are considered to reflect excellent agreement (Mchugh, 2012). We also applied this metric in our study.

Cohen’s kappa values in the study by Danker-Hopfe et al. (2009) showed good agreement for REM sleep, minimal agreement for stage 1 and moderate agreement for the other stages.

Shortly after a sleep scoring standard was established in 1968 (Rechtschaffen and Kales, 1968), attempts were made to develop algorithms for automated sleep staging (Itil et al., 1969; Larsen and Walter, 1970; Smith and Karacan, 1971; Martin et al., 1972; Gaillard and Tissot, 1973; Gevins and Rémond, 1987).

### Related Work

Martin et al. (1972) applied a simple decision tree using EEG and EOG data for scoring. A decision tree like algorithm was also used by Louis et al. (2004). Stanus et al. (1987) developed and compared two methods for automatic sleep scoring: one based on an autoregressive model and another one based on spectral bands and Bayesian decision theory. Both methods used one EEG, two EOG and an EMG channel. The EOG was needed to detect eye movements and the EMG to assess the muscle tone. Fell et al. (1996) examined automatic sleep scoring using additional non-linear features (correlation dimension, Kolmogorov entropy, Lyapunov exponent) and concluded that such measures carry additional information not captured with spectral features. Park et al. (2000) built a hybrid rule- and case- based system and reported high agreement with human scorers. They also claimed that such a system works well to score patients with sleep disorders.

One of the commercially successful attempts to perform automatic scoring evolved from the SIESTA project (Klosh et al., 2001). The corresponding software of the SIESTA group was named Somnolyzer 24x7. It includes a quality check of the data based on histograms. The software extracts features based on a single EEG channel, two EOG channels and one EMG channel and predicts sleep stages using a decision tree (Anderer et al., 2005). The software was validated on a database containing 90 patients with various sleep disorders and ∼200 controls. Several experts scored sleep in the database and Somnolyzer 24x7 showed good agreement with consent scoring (Anderer et al., 2005).

Newer and more sophisticated approaches were based on artificial neural networks (ANNs). Schaltenbrand et al. (1993) for example applied ANNs for sleep stage classification using 17 features extracted from PSG signals and reported an accuracy close to 90%. Pardey et al. (1996) combined ANNs with fuzzy logic and Längkvist et al. (2012) applied restricted Boltzmann machines to solve the sleep classification problem, to mention just a few approaches.

The methods mentioned above require carefully engineered features. It is possible to avoid this step using novel deep learning methods. ANNs in the form of convolutional neural networks (CNNs) were recently applied to the raw sleep EEG by Tsinalis et al. (2016). CNNs are especially promising because they can learn complex patterns and ‘look’ at the data in a similar way as a ‘real brain’ (Fukushima and Miyake, 1982). However, working with raw data requires a huge amount of training data and computational resources.

Sequences of epochs are considered by a human expert according to the scoring manuals. Therefore, we assume that learning local temporal structures are an important aspect in automatic sleep scoring. Temporal patterns have previously been addressed by applying a hidden Markov model (HMM) (Doroshenkov et al., 2007; Pan et al., 2012). In the last few years, recurrent neural networks (RNNs) have demonstrated better performance than “classical” machine learning methods on datasets with a temporal structure (Mikolov et al., 2010; Graves et al., 2013; Karpathy and Fei-Fei, 2015). One of the most common and well-studied RNNs is the Long-Short Term Memory (LSTM) neural network (Hochreiter and Schmidhuber, 1997). Such networks have been successfully applied to EEG data in general (Davidson et al., 2006) as well as to sleep data (Supratak et al., 2017).

Artificial neural networks using raw data revealed comparable performance as the best ANNs using engineered features and the best classical machine learning methods (Davidson et al., 2006; Tsinalis et al., 2016; Supratak et al., 2017; Chambon et al., 2018; Phan et al., 2018; Sors et al., 2018). See Section “Discussion” for more details.

The above-mentioned approaches were based on supervised learning. There have also been several attempts to perform unsupervised automatic sleep scoring in humans (Gath and Geva, 1989; Agarwal and Gotman, 2001; Grube et al., 2002) and in animals (Sunagawa et al., 2013; Libourel et al., 2015).

### Our Contribution

We implemented different machine learning algorithms, random forests (RF), feature based networks (LSTM networks) and raw-data based networks (CNN-LSTM networks) and trained and tested them in healthy participants and patients. We report all the Cohen’s kappa values (Cohen, 1960) of the different stages for the comparison of the performance the algorithms.

All our algorithms yielded high values of Cohen’s kappa of the data of healthy subjects. Performance on data recorded in patients was lower, but less so for ANNs. Including part of the patient data into the training improved performance on the patient data. This suggests that we would need even larger and diverse datasets to train an algorithm which can be applied reliably in practice. DNNs performed well even using only a single EEG channel, an interesting observation of our work.

## Materials and Methods

### Polysomnographic (PSG) Data

We trained and tested automatic sleep stage scoring algorithms on two datasets from two different laboratories.

**FIGURE 2:**
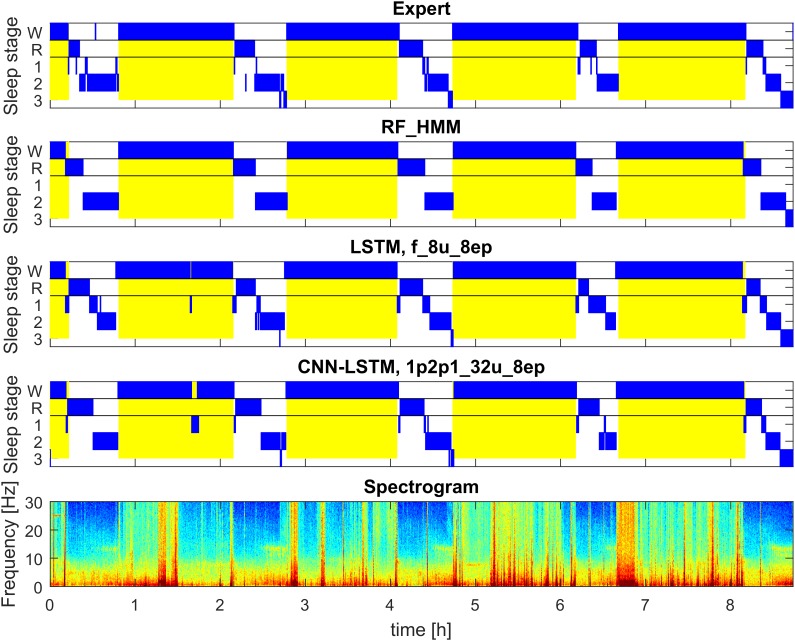
Example of automatic sleep scoring of MSLT data trained on a mixture of data of healthy participants and patients data (datasets 1 and 2; example of test set). Figure structure and abbreviations are analogous to Figure 1. Yellow background represents lights on.

The first dataset was comprised of 54 whole night sleep recordings of healthy participants. The second dataset consisted of 22 whole night sleep recordings and 21 recordings of a multiple sleep latency test (MSLT) in patients. The MSLT is routinely used to evaluate daytime sleepiness of patients. During this test a subject has four or five 20-min nap opportunities, which are separated by 1.5-h long intervals. An example of an MSLT hypnogram can be seen in Figure 2. Usually, only naps are recorded, but in our dataset, recordings were continuous over approximately 9 h and occasionally we observed sleep episodes in addition to the scheduled naps. In a standard setting these sleep episodes would have been missed. EEG channel C3A2, one myographic and two oculographic channels were used for analysis and classification.

#### Dataset 1: Healthy Subjects

Polysomnographic (PSG) recordings from a study investigating the effect of vestibular stimulation (Omlin et al., 2018). In total 18 healthy young males (20–28 years; mean: 23.7 years) were recorded. Three nights of sleep (8 h) were recorded in each subject. Two nights with motion (bed was rocked till sleep onset or for the first 2 h after lights off), and a control night without movement. Data were composed of 12 EEG channels, applied according to the 10–20 system, 2 EOG derivations, 1 submental EMG derivation, 1 ECG derivation and respiration signals (chest and abdomen). Recordings were performed with a polygraphic amplifier (Artisan, Micromed, Mogliano, Veneto, Italy). Sampling rate was equal to 256 Hz (Rembrandt DataLab; Version 8.0; Embla Systems, Broom Field, CO, United States). A high pass filter (EEG: −3 dB at 0.16 Hz; EMG: 10 Hz; ECG: 1 Hz) and an anti-aliasing filter (−3 dB at 67.4 Hz) were applied to the analog signals. The EEG derivations were re-referenced to the contra-lateral mastoids (A1, A2). Sleep stages (20-s epochs) were scored according to the AASM criteria (Iber et al., 2007). The study was performed in the sleep laboratory of the Institute of Pharmacology and Toxicology at the University of Zurich and was approved by the Institutional Review Board of the Swiss Federal Institute of Technology in Zurich (ETH Zurich).

#### Dataset 2: Patients

Data were recorded in patients with narcolepsy (23 patients) and hypersomnia (five patients) during a night of sleep (approximately 8 h) and during a MSLT (continuous recordings over approximately 9 h). We had to exclude some recordings due to bad signal quality. Thus, some patients contributed only with a night or a MSLT recording (Hypersomnia: 5 MSLT, 4 nights; Narcolepsy: 16 MSLT, 18 nights). Data were comprised of 6 EEG, 2 EMG, 2 EOG derivations and 1 ECG. Signals were recorded at a sampling rate of 200 Hz (polygraphic amplifier Grass Technologies AURA PSG). A high pass filter (EEG: −3 dB at 0.5 Hz) and an anti-aliasing filter (−3 dB at 50 Hz) were applied to the analog signals. Sleep stages (30-s epochs) were scored according to Rechtschaffen and Kales (1968). Movement time was not scored. To make sleep stages compatible with the first dataset, we merged sleep stages 3 and 4. Recordings were performed at the Sleep Disorders Center, Department of Clinical Neurophysiology, Institute of Psychiatry and Neurology in Warsaw, Warsaw, Poland. The study was approved by the Institutional Review Board of Institute of Psychiatry and Neurology.

Data in the two laboratories were recorded with different recording devices which resulted in different sampling rates and filter settings. Signals were resampled at 128 Hz (with applying appropriate anti-aliasing filters thus, leading to a similar low-pass filtering of the data) to accommodate data recorded at different sampling rates. We did not adjust the high-pass filtering because we did not expect it to have a big impact on classification performance. Another reason was that we consider it is important that our methods work with data recorded with equipment that differs between laboratories.

### Machine Learning: Classification

Machine Learning is a branch of computer science, which allows to learn properties of the data and solve problems without direct programming of the decision rules. The main approaches in machine learning are supervised and unsupervised learning (Bishop, 2016). In this work we used a supervised approach in order to solve the problem of classification (Bishop, 2016). Classification algorithms solve the problem of assigning labels to the data. They are trained with labeled data, the training set, to learn properties of the data and the corresponding labels [supervised machine learning (Bishop, 2016)].

In this work, we solved the classification problem by applying supervised machine learning algorithms. We followed two approaches, (1) classification based on features (RF and ANNs) and (2) classification based on raw data (ANNs).

#### Classification Based on Features

Polysomnographic signals are very complex, but they reveal certain patterns crucial for scoring by an expert. For example, waves of certain frequencies: sleep spindles (12–14 Hz), slow waves (0.5–4 Hz), alpha waves (8–12 Hz), theta oscillations (4–8 Hz) are very important to distinguish the different sleep stages. These measures can be easily quantified in the frequency domain. We applied classical spectral analysis (Welch, 1967) but also a multi-taper approach (Babadi and Brown, 2014) might be considered in particular when spectrograms are used as features. Other important markers of sleep stages such as rapid and slow eye movements, eye blinks and muscle tone can also be quantified. Such measures are called features and the process of their definition is called feature engineering. Using carefully engineered domain-specific features for machine learning systems has a lot of advantages: it requires a small amount of training data, is fast and the results are interpretable. Another approach based on deep learning, working with raw data, is described later.

##### Preprocessing and feature extraction

In a first step, we used spectrograms of the EEG instead of using the raw signal. It is well known that spectra capture the major properties of the sleep EEG and this way we were able to significantly reduce the dimensionality of our data. Power density spectra were calculated for 20-s epochs (30-s for patient data) using the Welch function in MATLAB (FFT; average of four or six 5-s windows; Hanning windows; no overlap; frequency resolution 0.2 Hz). Spectra were plotted and color-coded on a logarithmic scale (Figures 1, 2). Spectrograms were limited to the range of 0.8–40 Hz to reduce the dimensionality of the data matrix.

We used a set of 20 engineered features for the classification (see Supplementary Material for their definitions). They include among others power in different frequency bands and their ratios, eye movements, and muscle tone. We did not exclude any epochs (i.e., included artifacts), because we wanted to have a system, which is ready to work with the data with a minimal requirement of manual pre-processing. Moreover, epochs with artifacts contain useful information: wakefulness is almost always accompanied by movement artifacts and a movement is often followed by a transition into stage 1. Quantitative analysis, however, such as the calculation of average power density spectra requires exclusion of artifacts which can be achieved using simple algorithms (Malafeev et al., 2018).

We used two different approaches for the classification based on features: RF and ANNs.

##### Random forest (RF)

One of the classical methods to solve classification problems is based on decision trees (Morgan and Sonquist, 1963; Hunt et al., 1966; Breiman et al., 1984). Every node of a tree corresponds to a feature and a corresponding a threshold value. For a data vector which has to be classified, we traverse the tree by comparing a corresponding feature to the threshold of the node. Depending on the outcome of the comparison, we go to the left or to the right branch. Once we have traversed the tree, we end up in a leaf that determines to which class the data point belongs to.

Decision trees have certain limitations (e.g., overfitting) (Safavian and Landgrebe, 1991; Mitchell, 1997). Overfitting means that an algorithm learns something very specific of the training data and the classifier can no longer predict new data.

A way to overcome these limitations is to create an ensemble of trees: i.e., to build many trees, each based on a random subset of the training data (Ho, 1995; Breiman, 2001). A data point is classified by all trees and we can compute the probability of a data point belonging to a particular class by the fraction of trees which “voted” for this class. RF classifiers and similar recent tree-based technique demonstrated state-of-the-art results on a variety of problems (Laptev and Buhmann, 2014, 2015; Chen and Guestrin, 2016).

We implemented the RF to classify sleep stages based on feature vectors (20 components). We computed probability vectors for every epoch (20 or 30 s). Further we considered the local temporal structure of sleep as described above about time course learning. We applied a HMM (see Supplementary Material) and a median filter (MF) with a window of three 20-s or 30-s epochs to smooth the data.

##### Artificial neural networks (ANNs)

For a long time, researchers have been trying to build a computer model of a neuron (Farley and Clark, 1954; Rochester et al., 1956) and use such models for data classification (Rosenblatt, 1958). This research resulted in the development of multilayer neural networks (Ivakhnenko and Lapa, 1967) which are now denoted ANNs.

Artificial neural networks consist of interconnected neurons. Every neuron performs multiplication of input signals with parameters called weights, summed up and sent to the output. One can train ANNs by adjusting (updating) the weights (Goodfellow et al., 2016). This process of training is also called optimization. ANN training requires a function which quantifies the quality of the classification. Such a function is called the loss function or cost function. The loss function must be differentiable, otherwise it is not possible to compute the gradients. An example of a loss function is the mean square error. In our work, we used the cross-entropy loss function (De Boer et al., 2005). Cross-entropy loss is a good measure of errors of networks with discrete targets. Targets are the ground truth values given by an expert, in our case the sleep stages.

#### Deep Learning With Raw Data

Deep neural networks (DNNs), a specific type of ANNs, can learn complex models. Moreover, DNNs can automatically learn features and the feature engineering step can be omitted. Features can be learned using, for example CNNs (Fukushima and Miyake, 1982; Lecun et al., 1989; Waibel et al., 1989). DNNs usually show better performance than feature-based methods, but it comes at the price of an increased computational demand and such networks require more training data. However, DNNs require much less manual adjustments than feature-based methods and thus are easier to implement and maintain.

##### Convolutional neural networks (CNNs)

A particular type of DNNs are CNNs. They were initially developed for image recognition (Fukushima and Miyake, 1982; Lecun et al., 1989; Waibel et al., 1989). The main property of CNNs is that they perform a convolution of an input with a set of filters, which have to be learned. They were successfully applied not only for image recognition, but also in speech recognition (Abdel-Hamid et al., 2014), text analysis (Dos Santos and Gatti, 2014) and many other areas. Moreover, CNNs have already been successfully applied to various types of physiological signals, including wake EEG recordings (Cecotti and Graeser, 2008; Mirowski et al., 2008). The filters have a certain size. Given the one-dimensional nature of our data, a filter is a vector of a specific length. The filter slides with certain step called a stride across the input data.

Another specific type of layers we used was max-pooling. It takes the maximal value of the sliding window and helps to achieve local invariance. The max-pooling layer also has a specific filter size and a stride.

##### Residual networks

Residual networks (He et al., 2016) are a special kind of ANNs where layers are connected not only in sequential order but also with so-called skip or residual connections which jump over one or multiple layers. Gradients can vanish when networks have a lot of layers. Residual connections prevent this problem and make the training of networks more efficient and make it possible to train very deep networks with large numbers of layers.

### Learning Time Dependencies

Common machine learning algorithms consider every data sample independent from the previous ones. This is the case for RF classification and common ANNs. However, experts take information about previous epochs into account when they perform sleep scoring. Thus, it would be useful to consider some temporal information (structure) in the sleep classification algorithm.

As was mentioned in the introduction, sleep has not only a local but also a global structure, such as sleep cycles (Achermann and Tarokh, 2014). However, this global structure should not be taken into account while scoring (visual or automatic), as it might be different in pathology or during naps. Therefore, we limited the temporal memory of our models (see below), but the information of several previous epochs is still important to consider for sleep scoring. We assume that if we learn long sequences, it would bias the algorithm and such models would perform poorly on recordings where such patterns are not present, e.g., in the MSLT recordings (short naps of 20 min) or disturbed sleep.

We implemented the learning of temporal structures of sleep in two ways. First, we applied a HMM (Stratonovich, 1960) to smooth the output of the RF classification (see Supplementary Material for details) and by a MF with a window size of three epochs, a very simple yet efficient approach to smooth the data (see Supplementary Material).

As a second approach we implemented RNNs. RNNs receive their own output of the previous step as additional input in combination with the new data vector. Thus, RNNs take into account the temporal structure of the data. One of the most successful RNNs is the LSTM network (Hochreiter and Schmidhuber, 1997). RNNs can also use information about future epochs; in such a case they are called bidirectional RNNs. One of the main advantages of LSTM networks is its property to avoid vanishing gradients.

As mentioned above, the length of the input sequences should be limited to reasonably short time intervals. We limited our algorithms to learn patterns not longer than 8 (2.8 or 4 min), 32 (10.7 or 16 min), and 128 epochs (42.6 or 64 min). We dynamically formed batches of sequences: the beginning of each sequence was chosen randomly (i.e., sequences may intersect). This way more sequences may be used for training than by just taking them sequentially. For details about batches and their processing see Supplementary Material.

### Study Setup

#### Network Architectures

We considered two types of networks:

(1) Networks which used features as input (LSTM networks).
(2) Networks which worked with raw data and used convolutional layers before the LSTM networks (CNN-LSTM networks).

#### LSTM Networks

**FIGURE 3:**
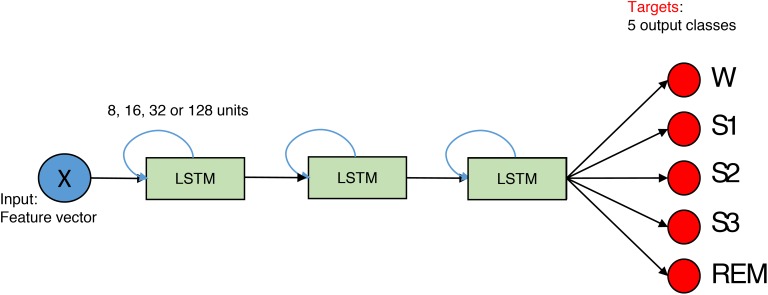
The structure of the network for feature-based classification. It is composed out of three layers. The size of the layer is 8, 16, 32, or 128 units. Blue arrows indicate that LSTMs are recurrent. *X* is the input data matrix – the matrix which contains features in columns and rows correspond to epochs. In case of the spectrogram as input, it corresponds to a transposed spectrogram. Red circles depict output neurons. Their output is compared to the expert labels (targets). Every neuron corresponds to certain sleep stage (W, Wake; S1, S2, S3, NREM sleep stages; REM, REM sleep).

We implemented a network with three hidden layers (Figure 3). Each layer consisted of 8, 16, 32, or 128 LSTM units, and we also applied one- and bi-directional layers resulting in a total of six network configurations.

#### CNN-LSTM Networks

**FIGURE 4:**
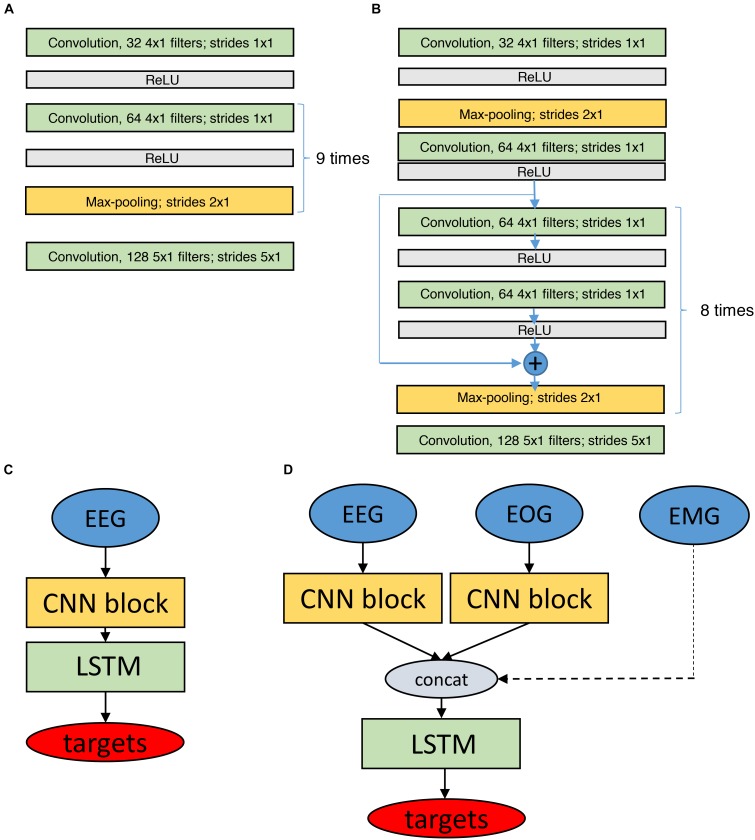
Structure of the networks for classification based on raw data. Networks have CNN and LSTM parts. **(A)** CNN block (11 layers) which is used to process raw EEG and EOG data. **(B)** Similar CNN block with residual connections (19 layers). **(C,D)** Depict the final network structures based on the modules depicted in **(A,B)**, using only EEG data **(C)** or EEG and EOG data as input (**D**, EMG input as dashed line as it did not have a CNN block). The EMG input was a preprocessed single value (power) per epoch. LSTM networks consisted of 2 bidirectional layers with 32 units each. There were batch normalization layers before, between and after LSTM layers. Batch normalization rescales inputs to make sure they all are in the similar range. Targets are the classified sleep stages. ReLU, Rectified Linear Unit, it is an activation function to transform the activation of a neuron.

We realized networks with 11 convolutional layers followed by two LSTM layers with 32 units (Figure 4).

We also used residual convolutional networks (19 layers) as outlined before, worked with different input signals (EEG, EOG, and EMG) and created separate CNN networks (CNN blocks in Figure 4) for every input (EEG, 2 EOG). The outputs of all blocks were concatenated and fed into the LSTM layers. There were two bidirectional LSTM layers. Each layer contained 32 LSTM units. There were batch normalization layers (Ioffe and Szegedy, 2015) before, between and after LSTM layers. Batch normalization layer rescales the input to make sure that all the values belong to the same range. We used separate CNN blocks for the two EOG channels because correlations between the EOG signals are important to distinguish the different types of eye movements. In case the EMG was included, only a single value (EMG power in the 15–30 Hz range) per 20- or 30-s epoch was considered. Thus, three input configurations were implemented: EEG only, EEG and EOGs, and EEG, EOGs and EMG (Figure 4) resulting in a total of seven network configurations.

### Optimization

Networks require training which is achieved by optimization. Optimization procedures have to find minima (in case of ANN local minima) of a loss function over the parameter space (weights of the network). Weights are commonly adjusted according gradients (backpropagation, see Supplementary Material for details about optimization and regularization).

Networks were implemented using the Keras package (Chollet, 2015) with Theano (Al-Rfou et al., 2016) and Tensorflow (Abadi et al., 2016) backends. The Theano backend was used to train our feature-based LSTM networks and the Tensorflow backend to train the raw data based CNN-LSTM networks. We worked with different backends because we first developed the feature based networks and running on a desktop computer and later with raw data based networks. These networks had to be trained on GPUs and for this only the Tensorflow (Abadi et al., 2016) backend was available.

### Training, Validation, and Testing

To avoid overfitting, we randomly split dataset 1 (healthy participants) into three parts: training (36 recordings, 70%), validation (9 recordings, 15%) and testing (9 recordings, 15%). The data were split according to participants, i.e., all three recordings of one participant were either in the training, validation or test set. We computed the cross-entropy loss and accuracy (De Boer et al., 2005) to assess convergence of the algorithms. These measures were computed on every training iteration for training and validation sets.

The idea was to train all our models using the training part of the data, then classify the data of the validation part and select only the best models for further confirmation of their performance on the test part. However, validation revealed that performance of the different models was very similar, thus, it was unclear whether their performance was really different. Therefore, we estimated the final performance of all algorithms with both the validation and test set. In addition, we used the whole second dataset (patients) as a test set, thus, assessing generalization of the approaches to datasets from another laboratory and to a different subject population (patients).

Further, we wanted to study how performance of the algorithms would benefit from the inclusion of patient data into the training set. We took the same training set of healthy subjects (36 recordings) and added patient data (19 recordings) to it, resulting in a training set of 73 recordings. The remaining patient data (10 MSLT recordings and 14 sleep recordings) were used for performance evaluation together with the test set of the healthy participants (9 recordings; a total of 33 recordings). Again, all data of one patient were assigned to the training or test set. For further details see Supplementary Material.

#### Performance Evaluation

To assess performance of our algorithms, we used Cohen’s kappa (Cohen, 1960) a metric accounting for the agreement by chance and thus for imbalanced proportions of different classes. Kappa is a number ≤ 1 (can be negative), with one reflecting ideal classification. Values higher than 0.8 are considered to reflect excellent agreement (Mchugh, 2012).

## Results

### Convergence of the ANNs

During the process of the training of ANNs we can observe an increase in the quality of the classification. To ensure that the network was sufficiently trained and further training would not bring additional benefit we computed cross-entropy loss and accuracy (proportion of correctly classified examples; see section “Materials and Methods” for details). Usually these metrics show an exponential saturation with increasing training time. After the accuracy or loss function have reached a plateau we can say that a network has converged. These types of curves are called learning curves (Pedregosa et al., 2011). We computed these curves on the training and validation datasets (50 training iterations in total).

All our feature-based LSTM networks showed good convergence when they were trained on the data of healthy participants (Supplementary Figure S3) and on a mixture of both datasets (Supplementary Figure S4; see Supplementary Material for the naming convention of the networks).

Learning curves for the ANN based on the raw data as input are depicted in Supplementary Figures S5, S6. Most of the networks showed good convergence (loss monotonously decreased, and accuracy increased to saturation). Some networks showed large fluctuations of loss and accuracy on the validation set: the network which has only a single EEG channel as input (1p_32u_8ep), the network which had EEG and EOG as input and eight epoch long sequences (1p2_32u_8ep), and the network with input comprised of EEG, EOG and EMG and 128 epoch long sequences (1p2p1_32u_128ep). The least smooth learning curves were observed in the network with residual connections. This network had the largest number of parameters and thus, more data and iterations might be needed to reach convergence. We expect that such networks to perform better if trained on an extended dataset.

### Classification Performance

The crucial information is how well the algorithms perform. As mentioned above we used Cohen’s kappa to measure the quality of the automatic scoring.

Figure 1 illustrates the hypnograms obtained with three selected algorithms (RF, LSTM, and CNN-LSTM) in comparison with the expert scoring. In general, performance of all algorithms was good capturing the cyclic structure of sleep. Slight differences to the human scorer were observed, e.g., longer REM sleep episodes with the 3-layer bidirectional LSTM network (Figure 1, panel 3).

Performance of our algorithms was initially assessed with the F1 score (Dice, 1945; Sørensen, 1948). But afterwards we switched to Cohen’s kappa (Cohen, 1960) because F1 scores are a biased measure of classification quality (Powers, 2014), which is a problem when comparing recordings with a different prevalence of the classes (sleep and MSLT).

#### Scoring of Healthy Participants

**FIGURE 5:**
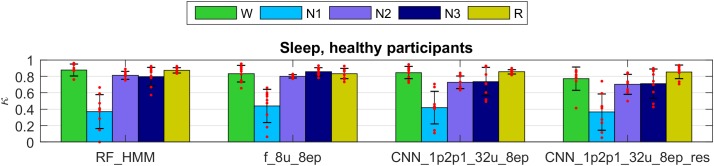
Cohen’s kappa of selected methods applied to the validation set of dataset 1 (healthy participants). The first 2 groups of bars represent feature-based classifiers (RF and LSTM network) and the last 2 groups CNN-LSTM networks based on raw data input. See Supplementary Material for the naming conventions of the algorithms. Mean values and standard deviations are depicted; red dots represent individual Cohen’s kappa values. W, wakefulness; N1–N3, NREM sleep stages; R, REM sleep.

The Cohen’s kappa computed on the validation part of the dataset 1 (healthy participants) are illustrated in the Figure 5 (only four selected methods; see Supplementary Tables S1, S2 for kappa of all algorithms, validation and test data): RF classification smoothed using HMM, one LSTM network trained on features, and two CNN-LSTM networks with raw data input, one of them included residual connections.

All four methods showed high performance for all stages except for the stage 1 (N1). Kappa of stage 1 was around 0.4 which we still consider a good result because it is comparable to the low human interscorer agreement of stage 1 (Danker-Hopfe et al., 2004; Danker-Hopfe et al., 2009; Penzel et al., 2013; Rosenberg and Van Hout, 2013).

The Cohen’s kappa of all methods evaluated on the validation part of dataset 1 are depicted in Supplementary Figure S7 (features) and Supplementary Figure S8 (raw data). Most networks performed similarly well on the validation set; those which included only a single EEG derivation as an input (Supplementary Figure S7, s_8u_8ep, spectrogram as input and Supplementary Figure S8, 1p_32u_8ep, raw EEG as input) showed slightly lower performance, probably since the EEG spectrogram or the raw EEG do not contain information about eye movements and muscle tone. However, this was the case in some recordings only, for other recordings the performance was very good. Interestingly, performance of these networks on the test set was much better (Supplementary Tables S1, S2). We assume that the validation set contained some recordings which were difficult to score using only a single EEG channel.

The network with input comprised of EEG, EOG, and EMG and 128 epoch long sequences (1p2p1_32u_128ep) had a low performance on both, the validation and the test set because of large random fluctuation of accuracy in the last training iteration. Ideally, we should have stopped training of this network earlier or trained it longer.

Networks with 16 and 32 units in a layer were inferior for the scoring of stage 1 than the network with only 8 units probably due to overfitting, although the difference was very small. These networks may show a better performance if trained with larger datasets. One-directional network predicted REM sleep a bit worse than bidirectional ones. The advantage of one-directional network is the possibility to work online. Surprisingly, classification with RF smoothed with simple MF or HMM worked almost as good as classification with ANNs (features and raw data).

#### Generalization to the Patient Data

**FIGURE 6:**
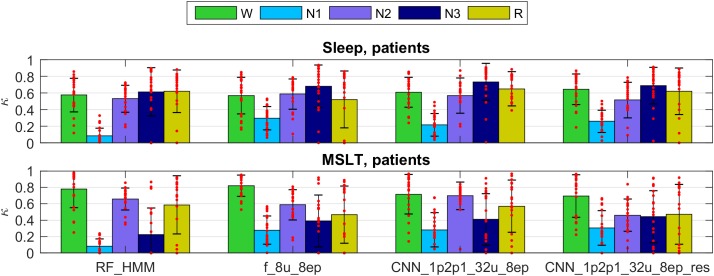
Cohen’s kappa scores for the same methods as in Figure 5 applied to the patient dataset. Note that the training did not include patient data. Top panel represents sleep recordings and the lower one MSLT recordings. Note that during MSLT recordings stage N3 is not always reached; such recordings were not taken into account when computing average Cohen’s kappa and standard deviations of N3. For details see Figure 5.

We validated our methods on dataset 2 (patients). The kappa values for selected methods are presented in Figure 6 (only 4 selected methods; see Supplementary Figures S9, S10 and Supplementary Tables S3, S4 for kappa of all algorithms used to classify patient data). Note that the data of the patient dataset were not used for the training at all.

The performance was somewhat lower for all classifiers applied to the sleep data of patients than in healthy participants and again lower for the MSLT data and kappa showed a large variance. Classification performance of stage 1 was worst for the RF classification in this dataset. Methods using only a single EEG signal as input (spectrogram or raw EEG channel as input) performed worse on the patient data.

We observed very low kappa scores in some recordings, mostly for stages 2, 3 and REM sleep in patients when the training data did not include patient data. Stage 2 was often confused with stage 1. We can explain it by different properties of sleep in patients. Their sleep was much more fragmented and disturbed. Thus, algorithms not trained with patient recordings may confuse stages 2 and 1. Kappa of stage 3 was very low mostly due to the low occurrence of deep sleep in patients, or its complete absence. Thus, small discrepancies led to low kappa values. Further, REM sleep was sometimes missed due to differences between patients and healthy participants. Sometimes REM sleep was falsely discovered. It happened because patients sometimes had a low muscle tone in wakefulness. Some of the false discovered REM sleep turned out to be true REM sleep missed by an expert (confirmed by visual inspection).

Algorithms based on the EEG only made most mistakes. Adding ocular channels to the input resulted in less mistakes and including included muscle tone also revealed the best performance.

When the networks were trained also using patient data the result have improved.

#### Networks Trained on the Data From Both Datasets

Next, we trained two networks and RF classification with a mixed training data consisting of healthy subjects (36 recordings) and part of the patient data (19 recordings; both sleep and MSLT data). We validated the models on the test part of the mixed dataset (healthy participant: 9 recordings; patients, 14 sleep and 10 MSLT recordings).

**FIGURE 7:**
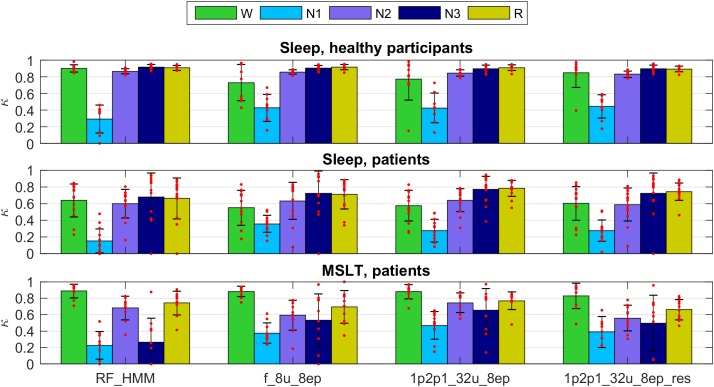
Cohen’s kappa for the methods illustrated in Figures 5, 6 trained on a mixture of data of healthy participants and patients data (datasets 1 and 2; applied to the test part of both datasets). Top: healthy subjects; middle: sleep recordings in patients; bottom: MSLT recordings in patients. For details see Figures 5, 6.

Figure 2 illustrates the hypnograms of a MSLT recording obtained with three selected algorithms in comparison with the expert scoring. In general, performance of all algorithms was good capturing the naps. Performance of four selected methods are illustrated in Figure 7, and of the other methods applied in Supplementary Figures S11, S12 and in Supplementary Tables S5, S6. Note, that we trained only two feature-based networks with the mixture of the two datasets. Training on the mixed data resulted in an improved performance on both patient data and data of healthy participants, kappa values increased and the variance got smaller.

As mentioned above the performance after training on both datasets was better than training only on data of healthy participants, which is not surprising. It is well known that sleep of the patients (narcolepsy and hypersomnia in our case) is quite different compared to healthy participants. Again, algorithms based on EEG, EOG, and EMG revealed reasonable kappa values for all recordings except for circumstances where some stages were not present in a recording, or only in very small amounts. This was often the case for stage 3 in almost all MSLT recordings and in some sleep recordings of the patients. Often discrepancies occurred at stage/state transitions. However, that is where mostly experts also disagree. Multiple expert scoring of the same recording would be needed to establish a “ground truth.” Occasionally, the EEG was contaminated by ECG artifacts leading to a lower classification performance. Thus, removal of ECG artifacts prior to classification might improve the performance.

## Discussion

### Comparison With Human Experts and Automatic Scoring of Other Groups

All our implemented methods yielded high Cohen’s kappa values (kappa around 0.8) for all stages when they were trained and validated on data of the same type of subjects, except for stage 1 (N1; kappa < 0.5). Stage 1 is known as a difficult stage to score.

Common measures of interrater agreement are accuracy and Cohen’s Kappa (Danker-Hopfe et al., 2004, 2009;Penzel et al., 2013; Rosenberg and Van Hout, 2013). Kappa values obtained with our models were comparable to the performance of human experts. Stage 1 was most difficult to score automatically and compares with the low interrater agreement (Danker-Hopfe et al., 2004, 2009; Penzel et al., 2013; Rosenberg and Van Hout, 2013).

Performance of the LSTM networks in our experiments were similar to the one of a recent study where a CNN was applied to EEG features (Tsinalis et al., 2016) and to Phan et al. (2018) who applied CNNs to spectral features of EEG, EOG, and EMG channels.

Our CNN-LSTM networks performed similar to the ones of recent studies which employed CNNs for sleep scoring based on a single EEG derivation (Sors et al., 2018) and on six EEG channels in conjunction with two EOG and three EMG channels (Chambon et al., 2018). Sors et al. (2018) used a large database to train their network. They reported Cohen’s kappa computed over all classes equal to 0.81. Our values were close to it, however, it is not possible to compare directly because we looked at each class separately. We consider it very important to know the kappa values for wake, NREM and REM sleep separately due to their unbalanced contribution.

Supratak et al. (2017) used a technique known as residual sequence learning which might improve the performance. We did not apply this approach but used residual connections and different signals as independent inputs in the convolutional part of the network which were concatenated as input to the LSTM part. We think this was beneficial for the performance.

Even though automatic scoring algorithms have shown reasonably high performance there is no consensus yet in the sleep community that they perform well enough to replace human scorers.

### Automatic Scoring Using Different Channels

Our study showed that it is possible to score sleep data with high classification accuracy using only a single EEG channel. We got slightly better results using 1 EEG, 2 EOG, and 1 EMG channel.

It is difficult to conclude which method works best due to the small differences in performance. We assume that four channels (1 EEG, 2 EOG, and 1 EMG) contain more information, but the risk of the data being noisy is also higher. We observed that a bad EMG signal reduced the performance of the algorithms. This was also observed by SIESTA team (Anderer et al., 2005). The authors reported that in some cases the use of the EMG was not optimal due to a bad signal quality, and in certain cases they substituted the EMG with the high frequency content of the EEG and EOG which increased the performance of their algorithm. Also Phan et al. (2018) showed that the use of EOG and EMG channels was beneficial and Chambon et al. (2018) reported that the use of multiple EEG channels increased the performance of automatic sleep scoring.

It was surprising to observe that neural networks can classify sleep, especially REM sleep, with high quality using only a single EEG channel. It is a very difficult task for a human scorer to distinguish REM sleep based on the EEG only. Experts rely on eye movements and muscle tone (Rechtschaffen and Kales, 1968; Iber et al., 2007). We think that presence of patterns such as sawtooth waves (Jouvet et al., 1960; Takahara et al., 2009) are important markers of REM sleep which help neural network to recognize this stage.

A note of caution regarding EEG channels: the signal amplitude is strongly dependent on the referencing and the scoring of SWS (N3) is dependent on an amplitude criterion (75 μV peak-to-peak) (Rechtschaffen and Kales, 1968; Iber et al., 2007). We and others (Chambon et al., 2018; Phan et al., 2018; Sors et al., 2018) used, as it is standard in the sleep field, EEG derivations referenced to the contralateral mastoid whereas a different referencing was used in other studies (Tsinalis et al., 2016; Supratak et al., 2017). Networks trained with specific referencing should not be applied to data recorded with a different reference system as in particular the amount of SWS (N3) will be affected due to the difference in signal amplitude.

### How to Measure Scoring Quality?

It is difficult to determine which method was superior based on our results. We think this is because most of our methods showed high performance based on the chosen evaluation metric and produced results comparable to human experts.

An issue is the fact that with F1 scores (Dice, 1945; Sørensen, 1948) and Cohen’s kappa (Cohen, 1960) we treat epochs independently not taking the temporal structure of sleep into account. Thus, we think such metrics are not the optimal way to assess different aspects of the quality of scoring. For example, visual inspection of our results has shown that quiet wakefulness at the beginning of sleep might be confused with REM sleep and sometimes the first often very subtle and short REM sleep episodes might be missed. Such misclassification often occurred when the EMG or EOG signals were corrupt or of bad quality. It almost does not affect F1 scores and kappa values but affects the structure of sleep. Thus, novel metrics to quantify the scoring quality shall be developed that take the temporal structure into account but not overestimating differences at transitions, e.g., the start or end of REM sleep episodes.

### Which Method Is the Best?

Despite the difficulties to select the best method as the performance was very similar, we see some trends. Neural networks of all types detected stage 1 better than RF classifiers. This was especially evident when we applied the methods to the second dataset (patients), which indicates a better generalization of neural networks.

The RF classification with HMM and MF smoothing was superior to the RF classification without smoothing, and the networks based on the raw data input tended to be superior to features based networks, in particular when they were applied to the data of another laboratory and to a different subject population.

Given that our results and those of other groups (Tsinalis et al., 2016; Supratak et al., 2017; Chambon et al., 2018; Phan et al., 2018; Sors et al., 2018) are very close to the performance of the human expert we think that future evaluations of automatic scoring shall be performed using the multiple expert scoring and some other metric than F1 score or Cohen’s kappa. An ideal metric shall take the temporal structure of sleep into account and treat sleep not as a set of epochs but as a set of sleep episodes. For example, a short REM sleep episode at the sleep onset does not affect F1 scores and kappa much, but might be of clinical significance. A good metric shall penalize such mistakes in scoring.

### Importance of the Training Data

An improvement of performance was achieved when the training was performed on a mixture of the two datasets, which suggests that one should train on as diverse data as possible to reach best performance. However, the models trained only on the first dataset (healthy participants) performed reasonably well on the second “unfamiliar” dataset (patients) showing a good generalization.

In case an electrode has high impedance, the signal might become very noisy. For example, as neural nets learned that a low muscle tone is required to score REM sleep, noisy, or bad EMG signals may deteriorate the performance considerably. The same holds for the EOG: if the signal quality is bad, then the algorithms may not be able to detect eye movements properly. These problems can be addressed by visual inspection of the signals before applying an algorithm and selecting the one working best with the available signals. It is also possible to develop tools for automatic examination of data quality and the subsequent selection of a corresponding algorithm.

Sometimes our models mistakenly classified epochs close to sleep onset as REM sleep, which is unlikely to occur in healthy subjects. A human expert most likely would not make such a mistake. This can be partially explained by the fact that we never presented the whole night to our neural networks and they could thus not learn that REM sleep is unlikely to occur at the beginning of sleep. Human scorers, however, have this knowledge. Some groups of patients, for example, those suffering from narcolepsy, often have REM sleep at the sleep onset, called sleep onset REM (SOREM) sleep episodes. Thus, it is important to be able to detect SOREM sleep episodes. They may occur also in healthy people in the early morning due to the circadian regulation of REM sleep (Sharpley et al., 1996; Mayers and Baldwin, 2005; Mccarley, 2007) or by experimental manipulation (Tinguely et al., 2014). They further may occur in sleep-deprived subjects, and in depressed patients, which are withdrawn from selective serotonin reuptake inhibitor (SSRI) medication (Sharpley et al., 1996; Mayers and Baldwin, 2005; Mccarley, 2007). Therefore, we did not introduce any priors preventing our algorithms from classifying epochs at sleep onset as REM sleep.

The main question, however, is how representative are the training data. We trained on healthy young participants and specific patients (narcolepsy, hypersomnia). Thus, it does not represent the entire spectrum of healthy subjects (form infancy to old age) and the patient population.

### Effect of the Length of the Sequence

We limited the length of the training sequences to 8 epochs but also tested the effect of 32 and 128-epoch long sequences. Networks trained on 128 epoch long sequences did not perform well when presented with unfamiliar datasets, i.e., they generalized worse. It might be the networks learned more global structures of sleep and thus did not perform well on recordings with different structures (MSLT, disturbed sleep, patients, etc.). We noticed that longer sequences led to less stage changes, i.e., more consolidated sleep stages than scored by experts. Thus, we think it is better to keep the length of the training sequence short (eight epochs).

### Room for Further Improvement

We see a lot of room for further improvement. The sleep scoring manual was first developed for the scoring of healthy sleep, and is also being used for sleep in different kind of patients and people under the influence of medication or drugs. The wake EEG can also be affected by substances (Von Rotz et al., 2017). Thus, we recommend extending the training data including data from different laboratories, different pathologies, age groups and so on. One can also try to use data augmentation to increase the robustness of neural networks.

A major limitation of our study was the expert scoring: it was performed by a single expert although different ones. We suppose, that performance would have increased if several scorers would have scored the same data and consensus scoring would have been used for the training of the models. Also, human scorers have difficulties with ambiguous data and interscorer variability results in part due epochs that are difficult to score with confidence (Younes et al., 2016).

We showed that our algorithms had a good generalization capability to the patient population, but the performance was not as good as with healthy subjects. One possible reason might be the different scoring epoch length. We used the conversion procedure which worked well for most epochs, but certain discrepancies may show up at transition phases. We think this might have limited the performance, especially when these data were used for training. It was a compromise we had to make. Ideally all the data would be scored with the same epoch length. Phan et al. (2018) used a different approach and converted 20-s epochs to 30-s epochs by including the 5 s before and after a 20-s epoch.

Another aspect concerns movement time resulting in an artifact. In our datasets it was not scored, and in the AASM manual (Iber et al., 2007) scoring of movement time was abolished, which in our opinion is not optimal. Movement time basically results in EEG artifacts and it is thus difficult to assign a particular sleep stage. We suspect that the performance of the algorithms would improve if movement artifacts would have been scored as a separate class. Similarly, every artifact scored as some stage of sleep causes problems as artifacts do not look like sleep and thus such issues are equivalent to mistakes in the labels presented to the machine learning algorithm.

Recent work with automatic scoring on a large dataset (Sun et al., 2017) revealed that increasing the size of the dataset improved the performance. In the case of Sun et al. (2017) saturation occurred at approximately 300 recordings in the training set. However, their approach was feature based. We expect that saturation will occur at much larger numbers of recordings in the training set in case of DNNs working with raw data.

We demonstrated that it is possible to reliably score sleep automatically in polysomnographic recordings using modern deep learning approaches. It was also possible to identify stage 1 and REM sleep as reliable as human experts. In general, our models provided high quality of scoring, comparable to human experts, and worked with data of different laboratories and in healthy participants and patients. Furthermore, it was possible to successfully score MSLT recordings with a different structure than night time sleep recordings. We demonstrated that the local temporal structure in the data is important for sleep scoring. Some of our methods may also be applied for the on-line detection of sleep and could thus be used with mobile devices or to detect sleep in a driving simulator.

## Ethics Statement

This study was carried out in accordance withthe recommendations of Review Board of the Swiss Federal Institute of Technology in Zurich, Switzerland (Dataset 1) and of the Institutional Review Board of Institute of Psychiatry and Neurology, Warsaw, Poland (Dataset 2), with written informed consent from all subjects in accordance with the Declaration of Helsinki. The protocol was approved by the Institutional Review Board of the Swiss Federal Institute of Technology in Zurich, Switzerland (Dataset 1) and by the Institutional Review Board of Institute of Psychiatry and Neurology, Warsaw, Poland (Dataset 2).

## Author Contributions

AM, DL, and PA designed the analyses. AM conducted the analyses. XO, RR, AM, PA, AlW, AdW, and WJ collected the data and performed initial analyses. SB and JB provided computational resources and consultations on the methods. AM and PA wrote the manuscript. All authors commented and accepted the final version.

## Conflict of Interest Statement

The authors declare that the research was conducted in the absence of any commercial or financial relationships that could be construed as a potential conflict of interest.
