# Supplementary material for: Automatic Human Sleep Stage Scoring Using Deep Neural Networks
Source: Front Neurosci. 2018 Nov 6;12:781. doi: 10.3389/fnins.2018.00781 (PMC6232272; doi:10.3389/fnins.2018.00781)
Supplement: Supplementary file 1 [file Table_1.docx]

Supplementary Material

# Automatic human sleep stage scoring using Deep Neural Networks

**Alexander Malafeev, Dmitry Laptev, Stefan Bauer, Ximena Omlin, Aleksandra Wierzbicka, Adam Wichniak, Wojciech Jernajczyk, Robert Riener, Joachim Buhmann and Peter Achermann***

*** Correspondence:** Peter Achermann: acherman@pharma.uzh.ch

# Definition of features

Twenty features were derived from the polysomonographic recordings (EEG, EMG, EOG). They are described in the following text. Matlab notations were used for their definitions if useful. All features were determined for consecutive 20- or 30-s epochs (epoch length used for sleep stage scoring). All signals were first resampled at 128 Hz to accommodate data recorded at different sampling rates.

The abbreviations of the features used are indicated in square brackets.

## Slow waves [slowWaves]

We counted the number of large amplitude slow waves per 20- or 30-s epoch [#/10 s]. They were detected according to Bersagliere and Achermann (Bersagliere and Achermann, 2010). The EEG signal was band-pass filtered (pass band: 0.5-2 Hz). Half-waves were detected as negative and positive deflections between zero-crossings. We counted only the half-waves with amplitudes larger than 37.5 µV according to the scoring rules. Slow waves are the most important marker of deep sleep. They can also be used to find REM sleep epochs due to the fact that they are absent in REM sleep (Rechtschaffen and Kales, 1968).

## EMG power [powEMG]

To quantify the muscle tone, EMG (electromyogram) power [µV2] in the 15-30 Hz range of consecutive 20- or 30-s epochs was determined (FFT, average of four or six 5-s epochs, Hanning window).

## EOG power [powEOG]

We recorded two EOG (Electrooculogram) channels: one of the left (LOC) and one of the right eye (ROC). Electrodes were placed above left corner of left eye and below right corner of the right eye (Figure S1). Both channels were referenced to the left mastoid (A1).

EOG power (1-5 Hz; [µV2]) of the combined EOG signal (i.e. the difference between the two signals, LOC-ROC; FFT, average of four or six 5-s epochs, Hanning window) of consecutive 20- or 30-s epochs was computed.

The EOG is caused by the movement of the eyeball (Young and Sheena, 1975). The eyeball is a dipole (Du Bois-Reymond, 1848), therefore rotations of the eyeball cause changes of the electrical potentials. The electrodes were placed in such a way that eye movements cause anticorrelated changes in the two channels. That is the reason why the difference of the two channels made eye movement-related changes more prominent and reduced the noise. EOG electrodes also pick up brain activity especially during slow wave sleep. This results in the appearance of oscillations similar to eye movements during slow wave sleep. In order to prevent the confusion between eye movements and slow waves we used the ratio powEOG/Delta to capture the occurrence of eye movements (see below).

## Frequency bands [Delta, Theta, Alpha, Spindles, Beta, Gamma]

EEG power [µV2] in different frequency bands correlate with sleep stages (Aeschbach and Borbély, 1993) and thus can be used to discriminate between the different stages. For example: delta power is elevated in deep sleep (Lessard and Paschall, 1970;Borbély et al., 1981), alpha activity appears during relaxed wakefulness with closed eyes in a majority of subjects (Berger, 1929), and sleep spindles are present in stage 2 (Rechtschaffen and Kales, 1968).

We computed EEG power density spectra (FFT, average of four or six 5-s epochs, Hanning window) for consecutive 20- or 30-s epochs and determined power in the following frequency bands (in Hz):

Delta: 0.8-5.0; Theta: 5.0-8.6; Alpha: 8.6-12.0; Spindles: 11.0-15.0; Beta: 16.0-30.0; Gamma: 30.0-40.0

We also used combinations of power in those frequency bands (Louis et al., 2004):

Delta.*Alpha)./(Beta.*Gamma)

Theta.^2./(Delta.*Alpha)

As mentioned above, powEOG/Delta was used to quantify the presence of eye movements.


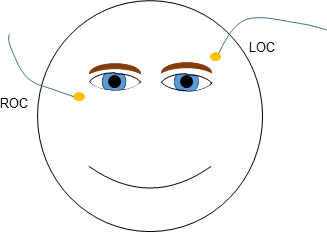


**Figure S1.** Placement of the EOG electrodes above the outer corner of the left eye and below the outer corner of the right eye. Electrodes were referenced to the left mastoid (A1).

## Brain rate (center frequency) [brain_rate]

We computed the “brain rate” [Hz] (Pop-Jordanova and Pop-Jordanov, 2005) as weighted sum of frequency values with weights equal to the relative power density in the corresponding frequency bin. It was computed in the frequency range: 0 - fs/2 Hz; fs sampling rate. Brain rate was reported to be a good measure of mental arousal (Pop-Jordanova and Pop-Jordanov, 2005). Brain rate can be computed using the BioSig package (Schlögl and Brunner, 2008) .

We computed it using following Matlab code:

faxis = 0:df:fs/2; % frequency range

brain_rate = (faxis*Pspec)./(sum(Pspec));

where Pspec is a spectrogram (matrix) with the size equal to [Number of epochs, Number of frequency bins] and df the frequency resolution (0.2 Hz in our case).

## Spectral Edge Frequency (SEF) [SEF90, SEF50, SEFd]

Spectral Edge Frequency (SEFxx [Hz]) (Drummond et al., 1991) is the frequency where xx percent of the power in the spectra is located below SEFxx. We abbreviate xx in percent, i.e. SEF50 denotes the frequency which divides the power density spectra in two equal parts, SEF90 the frequency which divides power density spectra in a lower part containing 90 % of the power and an upper part with 10 % of the power.

SEFxx was computed for consecutive 20- or 30-s epochs.

We used SEF50, SEF95, and their difference SEFd = SEF95-SEF50 as features. All values were computed in the frequency range of 8-16 Hz (Imtiaz and Rodriguez-Villegas, 2014) .

## Slow eye rolling [SEM]

Slow rolling eye movements occur at the transition from wake to sleep and during stage 1 (Rechtschaffen and Kales, 1968;Ogilvie et al., 1988). We implemented an algorithm developed by (Magosso et al., 2006). The method is based on a wavelet decomposition (10 levels; Daubechies wavelet of order 4 as mother wavelet). After performing the decomposition, we computed the function composed of the decomposition coefficients and thresholded them in order to detect slow eye rolling events.

We computed the amount of SEM events per 20- or 30-s epoch [#]. The input signal was computed as the difference between the LOC and ROC channels (LOC-ROC).

## Eye blinks and Rapid Eye Movements (REMs) [blinks_w, rem_w, eog_art]

Eye blinks are important because they occur only during wakefulness. That is the reason why we expected that eye blinks would be a useful feature to discriminate between wake, stage 1 and REM sleep. Rapid eye movements occur during REM sleep; therefore, this feature would be useful to discriminate REM sleep from other stages.

We noticed two distinct types of eye blinks (Figure S2). In most cases we observed a strong deflection in left EOG (LOC; electrode above eye; Fig. S1) and only a minor anticorrelated deflection in the right EOG (ROC; electrode below eye; Fig. S1). In some cases, we registered anticorrelated deflections of nearly equal amplitude in both channels.

Spontaneous blinks cause small movements of the eyeball, in combination with muscle activity it produces deflection in the EOG (Iwasaki et al., 2005). Saccadic eye movements were shown to be caused by the activity of the extraocular muscles (Carl et al., 2012). However, this does not fully explain the asymmetric deflections observed in LOC and ROC.

The LOC channel registers the activity caused by rotation of the eyeballs (dipoles) and of the muscles located above the eye and the ROC channel eyeball rotation and activity of the muscles below the eye (see Figure S1). Note that this asymmetric positioning of the EOG electrodes is crucial.

Eye blinks have a characteristic symmetric shape and their duration is short. We performed continuous wavelet transform with 32 levels of the LOC and ROC signals. We chose a Mexican hat wavelet because the shape of this wavelet is close to the shape of an eye blink.

coef_L=cwt(LOC,1:32,'mexh'); % entire night

coef_R=cwt(ROC,1:32,'mexh');

Then we sum all the coefficients and get two signals

wl = sum(coef_L(:,:)); % entire night

wr = sum(coef_R(:,:));

wl, wr are vectors (samples). The next step was to find peaks in wl (corresponding to LOC). We selected peaks with minimal height of 4000 separated by at least 0.2 s. (Note that function findpeaks was introduced recently in Matlab; we used version 2015b).

[wl_peak_amp, wl_peak_pos, wl_peak_widths] = findpeaks(wl, 'MinPeakHeight',4000, 'MinPeakDistance', round(0.2*fs)); % fs: sampling rate

Then we selected only the peaks with an amplitude ratio wl/wr smaller than -2. This condition ensures that we reject positively correlated deflections and requires that at least a minor anticorrelated deflection in ROC is present, which is usually the case for eye blinks. A second condition was the following – the ratio of the amplitude to the width of the peak should be > 150 samples (approx. 1 s) as we only need to consider narrow peaks because eye blinks are short lasting events.

Following Matlab code implements two conditions mentioned above:

ndx1 = wl_peak_pos(find(wl(wl_peak_pos)./wr(wl_peak_pos)<-2.0));

ndx2 = wl_peak_pos(find((wl(wl_peak_pos)./wl_peak_widths)>150));

blnkpos = intersect(ndx1, ndx2);

The sum of detected eye blink events in consecutive 20- or 30-s epochs [#] forms a feature for the classifier (blinks_w).

A next important step is to detect rapid eye movements and saccadic eye movements. These events have similar amplitude as eye blinks (Figure S2; green rectangle). They are characterized by a very steep initial deflection followed by a slow recovery. The deflections in LOC and ROC are strongly anticorrelated and have similar amplitudes. Since there is a step-like change we used a Haar wavelet to capture it.

We performed continuous wavelet transform (Haar wavelet) with 32 levels of the LOC and ROC signals and summed up of the coefficients.

coef_L=cwt(LOC,1:32,'haar');

coef_R=cwt(ROC,1:32,'haar');

wl = sum(coef_L (:,:));

wr = sum(coef_R(:,:));


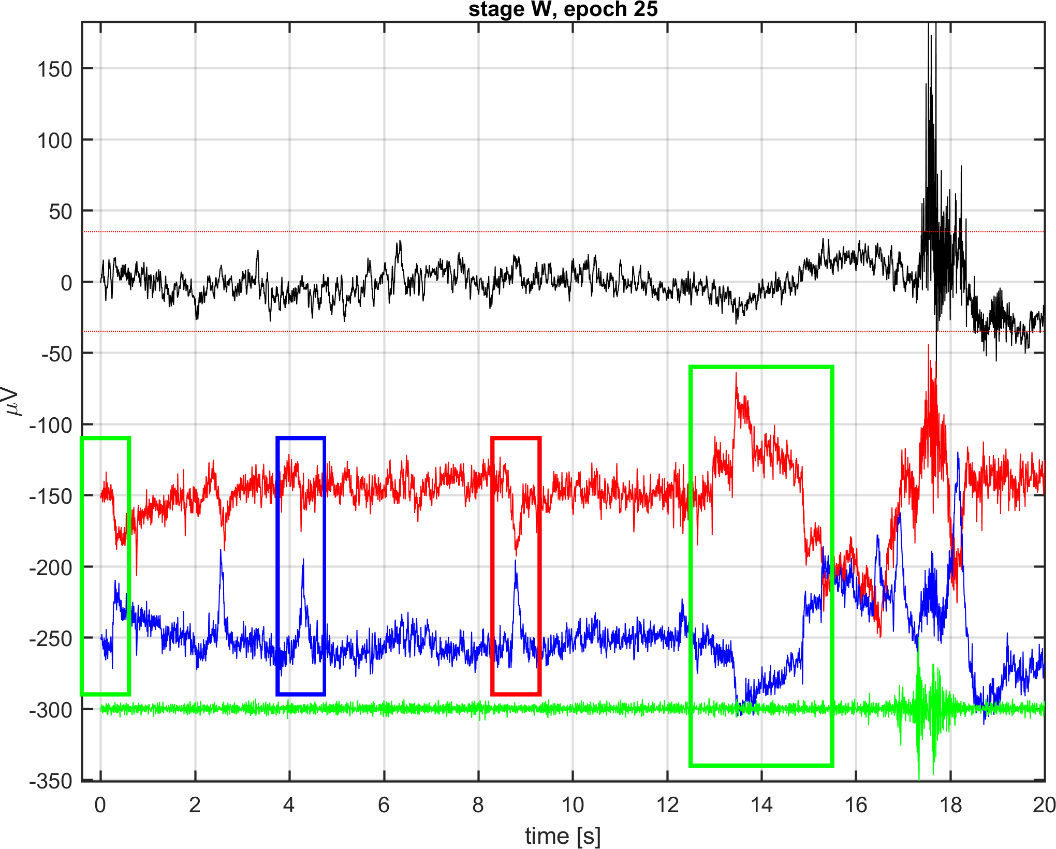


**Figure S2.** Example of one 20-s epoch of wakefulness. Black: EEG derivation C3A2 (red lines indicate ± 37.5 μV); red: right EOG (ROC, Fig. S1); blue: left EOG (LOC); green: chin EMG. Two types of eye blinks (blue and red rectangles) and a saccadic eye movement (green rectangle) are illustrated. Moreover, between 15 and 20 s a movement artifact occurred affecting all channels.

We also computed the correlation between LOC and ROC (Cor) on a sliding window. Window length was 1/8 s; moving step 1 sample.

Our main signal was a multiplication of wl, wr and (1-Cor) with normalizing constant of 1/200000. This signal is high when both wl and wr are high and anticorrelated.

Following Matlab code performs the computation:

wlwr = wl.*wr/200000.*(Cor'-1);

Then we select the peaks in wlwr:

[wlwr_peak_amp, peak_pos, peak_widths1 ] = findpeaks( wlwr, 'MinPeakHeight',10, 'MinPeakDistance', round(0.05*fs));

We required a minimal height of 10 and minimal distance between peaks of 0.05 s. Saccadic eye movements can occur very quickly one after another. That is the reason we have chosen such a short interval.

The next step was to filter out the peaks which correspond to rapid and saccadic eye movements.

We required a ratio of amplitudes between wl and wr of -0.3 and -1.7. Ideally it should be equal to -1 but, it may vary depending on the electrode position and signal quality.

Moreover, the ratio of the amplitude of wlwr to the width of the peak should be >1 to make sure that we do not confuse REMs with artifacts and slow eye movements. We also constrained the width of the peak. It had to be wider than 5 samples. This is very short but filters out artifacts (spikes).

ndx11 = peak_pos(find(wl(peak_pos)./wr(peak_pos)<-0.3));

ndx12 = PKpos(find(wl(peak_pos)./wr(peak_pos)>-1.7));

ndx1 = intersect(ndx11, ndx12);

ndx2 = peak_pos(find((wlwr(peak_pos)./peak_widths1)>1.0));

ndx3 = peak_pos(find(peak_widths1>5));

ndx23 = intersect(ndx2, ndx3);

rempos = intersect(ndx1, ndx23);

The number of detected REMs in consecutive 20- or 30-s epochs [#] formed a feature (REM_w).

Note that the thresholds for these two methods were derived based on our experience and common sense. We think that quantitative adjustment of the thresholds might improve the performance of eye movement detection. Note that the choice of the wavelets is a very strong prior and a crucial parameter of the methods.

We also used a feature EOG artifact (eog_art) in order to detect epochs where EOG channels were contaminated with artifacts. This feature is the amount of samples in LOC and ROC exceeding an absolute value of 350 µV.

# Feature vector

The final feature vector (20 components) is composed of the above defined features:

features_names = {'slowWaves', 'EMG', 'EOG/Delta','Spindles', 'Delta', ...

'Theta', 'Alpha', 'Beta', 'Gamma', ...

'Alpha/Theta', 'Beta/Theta', 'Alpha/Delta', 'Delta/Theta', ...

'(Delta*Alpha)/(Beta*Gamma)', 'Theta^2/(Delta*Alpha)', 'Brain rate', ...

'blinks_wav', 'rem_wav', 'SEM', 'eog_art'};

features = [slowWaves, powEMG, powEOG./Delta, Spindles, Delta, Theta, ...

Alpha, Beta, Gamma, Alpha./Theta, Beta./Theta, Alpha./Delta, ...

Delta./Theta, (Delta.*Alpha)./(Beta.*Gamma), Theta.^2./(Delta.*Alpha),...

Brain_rate', blinks_w', rem_w', SEM', eog_art' ];

Following transformation was applied:

ndx = [2:16];

features = log(log(features +1)+1);

features (:,ndx) = log(features (:,ndx)+1);

The original data were extremely skewed and we reduced the skewness by this transformation. We did not expect that a monotonous transformation would affect the random forest algorithm, but it might affect artificial neural networks.

# Taking the temporal structure into account by a Hidden Markov Model (HMM)

This model assumes that the system has a hidden state. This hidden state changes with certain probabilities, which are called transition probabilities. Transition of the system into the new state depends only on the current state (that’s why it is called Markov model), i.e. the system has memory only of one step (20- or 30-s epoch). The matrix of transition probabilities is called a transition matrix. As we cannot observe the hidden state directly, thus, it is referred to as a hidden state. Instead we can measure an observable variable. In our case the hidden state would be the sleep stage, whereas the observable variable is the probability vector resulting from the RF algorithm. We computed the transition matrix from the training set and applied the Viterbi algorithm (Viterbi, 1967) to infer the most probable sequence of stages. We employed RF and the Viterbi algorithm implementations of MATLAB version 2015a. We assumed uniform prior probabilities of all classes for RF meaning that without any information about the epoch the classifier would predict any of the classes with equal probability.

# Optimization of networks

## Gradient descent

One of the well-known algorithms of optimization is the gradient descent (GD). GD is based on the idea that if one moves along the maximal gradient in small steps one will end up in a minimum of a function.

In order to compute the gradients in the network, specific algorithms are used. The most widely applied algorithm for computing weight gradients of the neural networks is backpropagation (Werbos, 1974;Werbos, 1994). After the gradients are computed they are used to adjusts the weights accordingly (Bishop, 2016).

The problem of the gradient descent algorithm is that it requires the whole dataset to calculate the gradients. There are some tricks which help to reach the minimum faster. The algorithm still can converge to the optimum even if we use only one randomly selected training example from the training set to compute the gradients. The convergence happens much faster. On the other hand, such methods result in large fluctuations of the gradients. This method is called stochastic gradient descent (Bishop, 2016).

Usually gradients are computed over several data points to reduce fluctuations. These sets of data points are called batches. First, we need to split the whole training set into batches (see below).

When we have gone through all batches we have accomplished one training iteration (usually it is called training epoch, but we call it iteration to avoid confusion with the scoring epochs of the EEG data).

Another trick is to carry over some gradient from previous steps, i.e. to add momentum (Sutskever et al., 2013). It helps to reduce fluctuations of the gradient. One can imagine it with a very simply analogy: When you ski down the mountain you don’t change your direction on every small bump, you have a momentum directed towards the valley. There are several different ways to add the momentum to a gradient.

We trained the networks using the Adam (Adaptive moment estimation) (Kingma and Ba, 2014) algorithm with Nesterov momentum (Nesterov, 1983).

We also clipped the gradients: its norm could not be larger than 1. We used clipping only for the raw data based networks. Gradient clipping prevents gradients from becoming too large. If gradients become too large the convergence usually does not occur. This phenomenon is called explosion of gradients.

## Regularization

Regularization is needed to prevent overfitting. Different approaches might be applied. The simplest one is to penalize the weights. The most common approach used for neural networks is the dropout regularization.

We used both recurrent and non-recurrent dropouts (Hinton et al., 2012;Srivastava et al., 2014). Dropout regularization switches off certain neurons during training. It is considered to be an efficient regularization method for neural networks (Srivastava et al., 2014). The value of both types of dropouts in our networks was equal to 0.25. It means that 25% of the neurons were randomly switched off at each iteration.

The number of epochs of all classes (sleep stages) in the data is unequal. Moreover, the distribution of epochs of the different classes might differ between the training and test data. Thus, we assigned a weight to every class. In this way, every class contributed equally to the loss function as if there were equal amounts of epochs of all classes in the training data. The weight of a class X was equal to the ratio of the frequency of the most frequent class (in our case S2) to the frequency of class X. Frequencies and weights were computed within a batch.

## Batches

Our batches consisted of a specific number of sequences, each of them 8, 32 or 128 epochs long. The number of sequences in a batch was adapted to keep amount of data per batch similar for different sequence lengths.

For both types of networks (features and raw data) we applied the following parameters (note that in this context a sample is a sequence of scoring epochs):

samples_per_batch – number of sequences in one batch

samples – number of sequences sampled from each recording from sample_files

sample_files - number of files of the training set randomly chosen for every training iteration.

Thus, number of batches in every training iteration was (sample_files*samples)//samples_per_batch. “//” means integer division.

For training of the LSTM models on every training iteration we sampled (512 or 32) sequences with the corresponding length out of each recording. Samples for each batch were chosen randomly out of this subset. We chose sample_files = 36; samples_per_batch=512 or 32; samples = samples_per_batch.

For the CNN-LSTM models we had following numbers:

8-epoch long sequences: samples_per_batch = 100

32-epoch long sequences: samples_per_batch = 40

128 epoch long sequences: samples_per_batch = 10

Variable “Sample_files” was set to 16 and “samples” was set to 200 for all cases except the network with 128 epoch long sequences. For the latter network we set Samples = 100 due to the memory restrictions. Note that we used a random subset of the training data in each training iteration.

Thus, on every training iteration we randomly chose 16 recordings (sample_files) from the training set and sampled 200 or 100 sequences (samples) from each recording. Even though each training iteration did not contain all the training data (only 16 recordings), overall the networks were trained using the complete training set.

Even though we kept the amount of epochs per batch constant, the overall number of epochs per training iteration was proportional to the length of the input sequence.

## Training and validation

We used three machine learning approaches: random forests (RF) based on features, feature based networks (LSTM) and raw-data based networks (CNN-LSTM). We first trained all algorithms on the dataset 1 comprised of healthy participants (36 recordings which is approximately 70 % of 54 recordings) and validated them on the validation part (9 recordings which is approximately 15 % of 54 recordings) and test part (9 recordings) and on dataset 2 (patients, 43 recordings). In the next step, we trained all models using a mixture of the two datasets (55 recordings: 36 healthy sleepers and 19 patients) and validated on the mixed validation set (33 recordings: 9 nights of healthy subjects, 14 nights of sleep in patients and 10 MSLT recordings of patients). The idea was to test whether our models are transferable to datasets from another laboratory and to a different subject population (patients).

A difficulty in using a combination of both datasets for CNNs was the fact that sleep stage scoring was performed with a different epoch length (20 and 30 s) in the two datasets. We overcame this problem by converting the labels of the second dataset scored with 30-s epochs. We represented every 30-s epoch as three dummy 10-s epochs, all of them having identical labels. Then we reorganized the whole night in sequences of 20-s epochs consisting of two dummy epochs. Every such 20-s epoch was labeled according to the last (second) dummy epoch of this 20-s epoch.

# Naming conventions of algorithms

## RF classification

RF stands for Random Forest, RF_HMM means that the classification was smoothed using a HMM (see above), and RF_MF indicates smoothing with a moving median filter of length 3 (three 20- or 30-s epochs).

## LSTM networks

The structure of the networks was encoded in the name <f/s>_<un>u_<en>ep: <f/s> specifies the input of the neuronal network, with ‘f’ features and ‘s’ spectrograms of the EEG. <un> reflects the amount of LSTM units per layer (always 3 layers). <en> codes the length of the sequence used for training. If ‘1_dir’ follows at the end, it indicates that the network was unidirectional, otherwise it was bidirectional, i.e. it had information from the future for classification.

For example, f_16u_8ep means features as input, 16 units in each of the 3 layers, a sequence length of 8 epochs was used for training, and it was a bidirectional network. Recurrent activation functions of the LSTM were sigmoid and activation functions of LSTM were tanh.

## CNN-LSTM networks

The structure of the network was encoded in the name <input>_<un>u_<en>ep: <input> can be “1p” – a single raw EEG channel as input; “1p2” – a raw EEG and two raw EOG channels as input; “1p2p1” – same as previously and additionally EMG (muscle tone) as input; “p” stands for plus. <un> indicates the number of LSTM units per layer (always 2 layers). <en> codes the length of the sequence used for training. If ‘res’ follows at the end, it indicates that the network had residual connections.

For example, 1p2p1_32u_8ep_res means raw EEG, EOG and muscle tone as input, 32 LSTM units per layer, 8 epoch sequence length for training, and residual connections. Recurrent activation functions of the LSTM were sigmoid and activation functions of LSTM were tanh. Activation function of all convolutional layers was ReLU.

# Training and validation


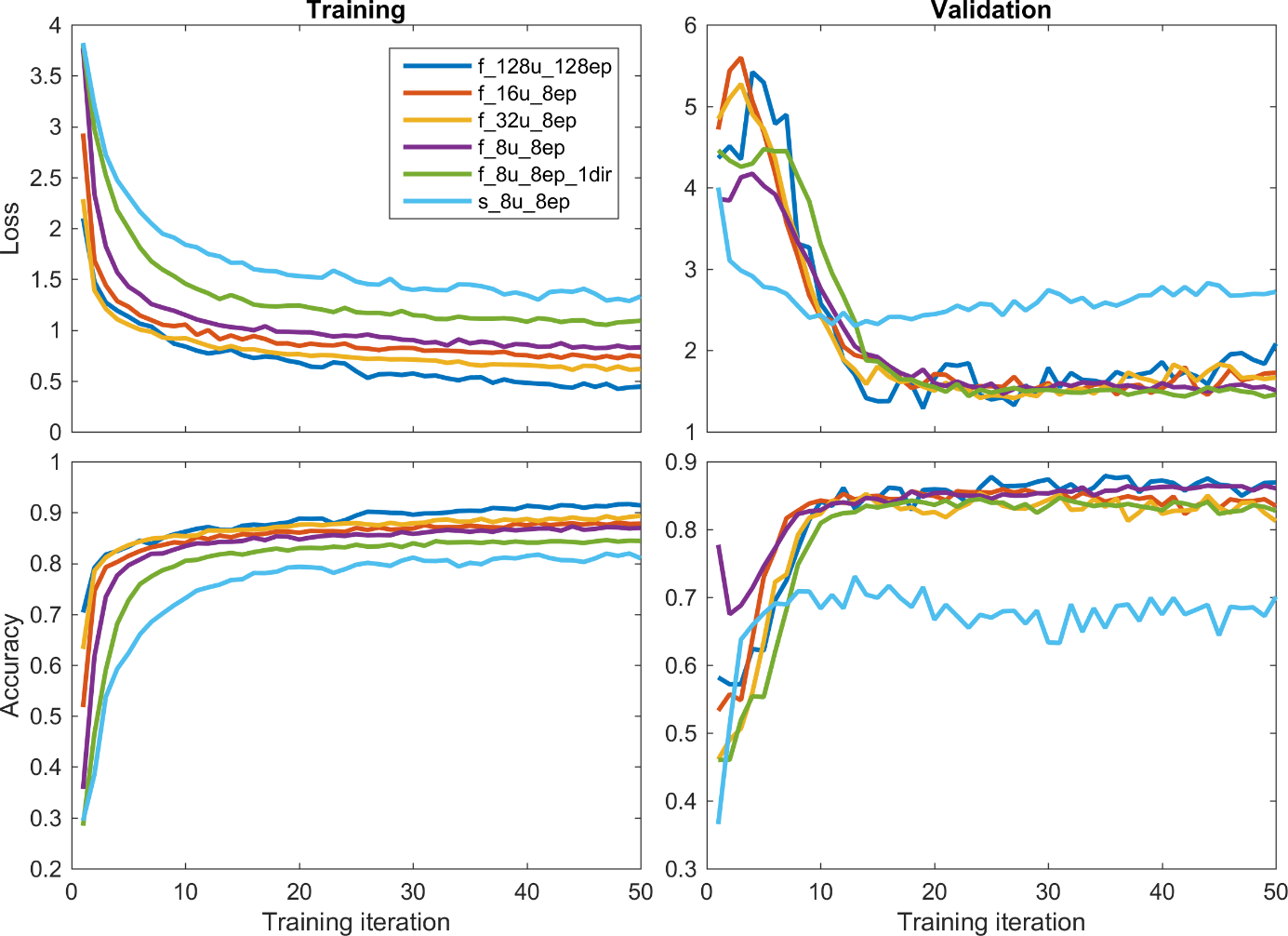


**Figure S3.** Learning curves of LSTM neuronal networks trained on dataset 1 (healthy participants). The networks were trained for 50 epochs (iterations, these epochs are not related to scoring epochs). The structure of the network was encoded in the name (see supplementary material for the naming convention). Left, loss and accuracy computed on the training data, right: on the validation data.


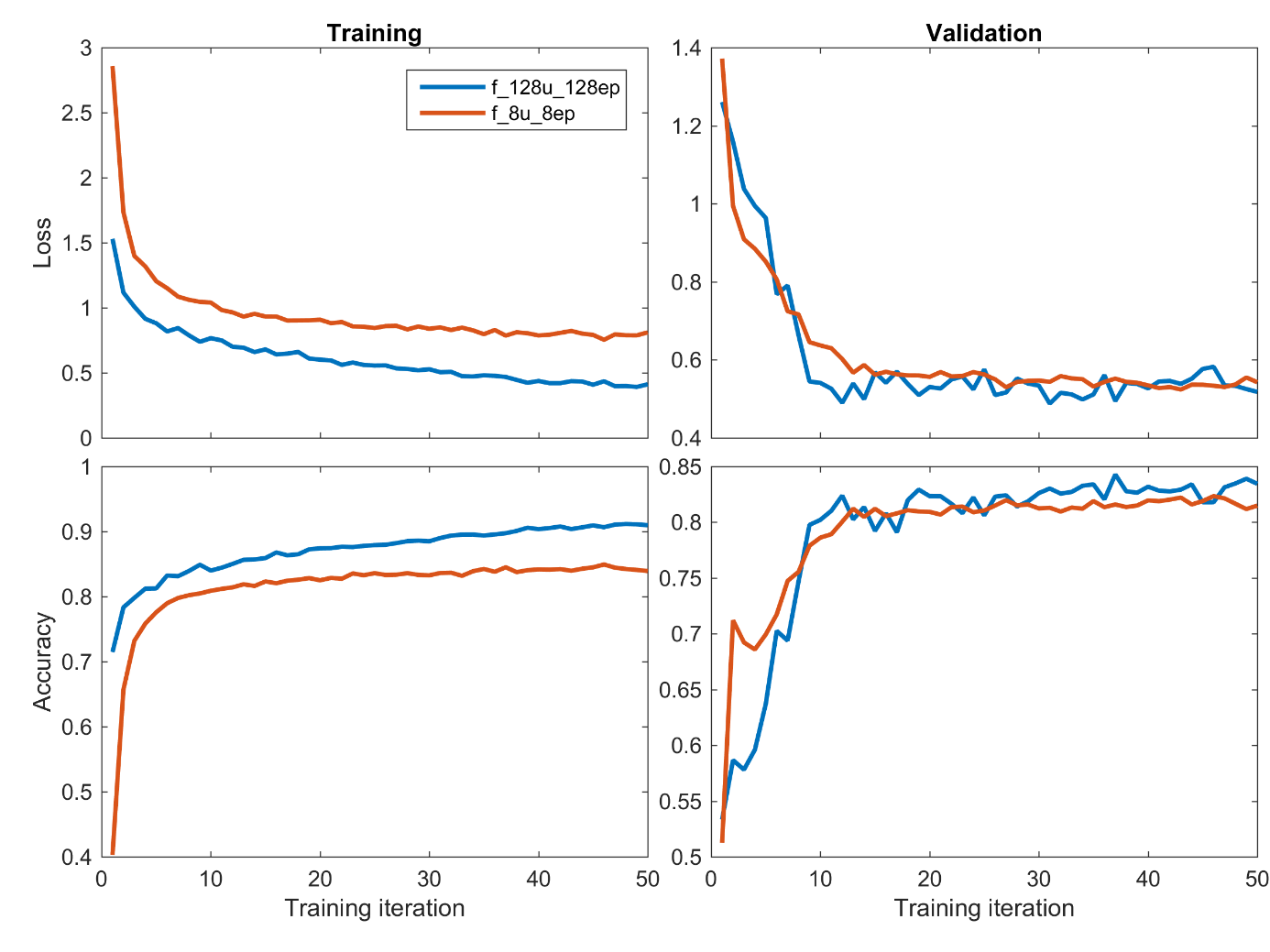


**Figure S4.** Learning curves of LSTM neuronal networks trained on a mixture of healthy subjects and patients (datasets 1 and 2). For details see Figure S3. Only 2 networks were trained.


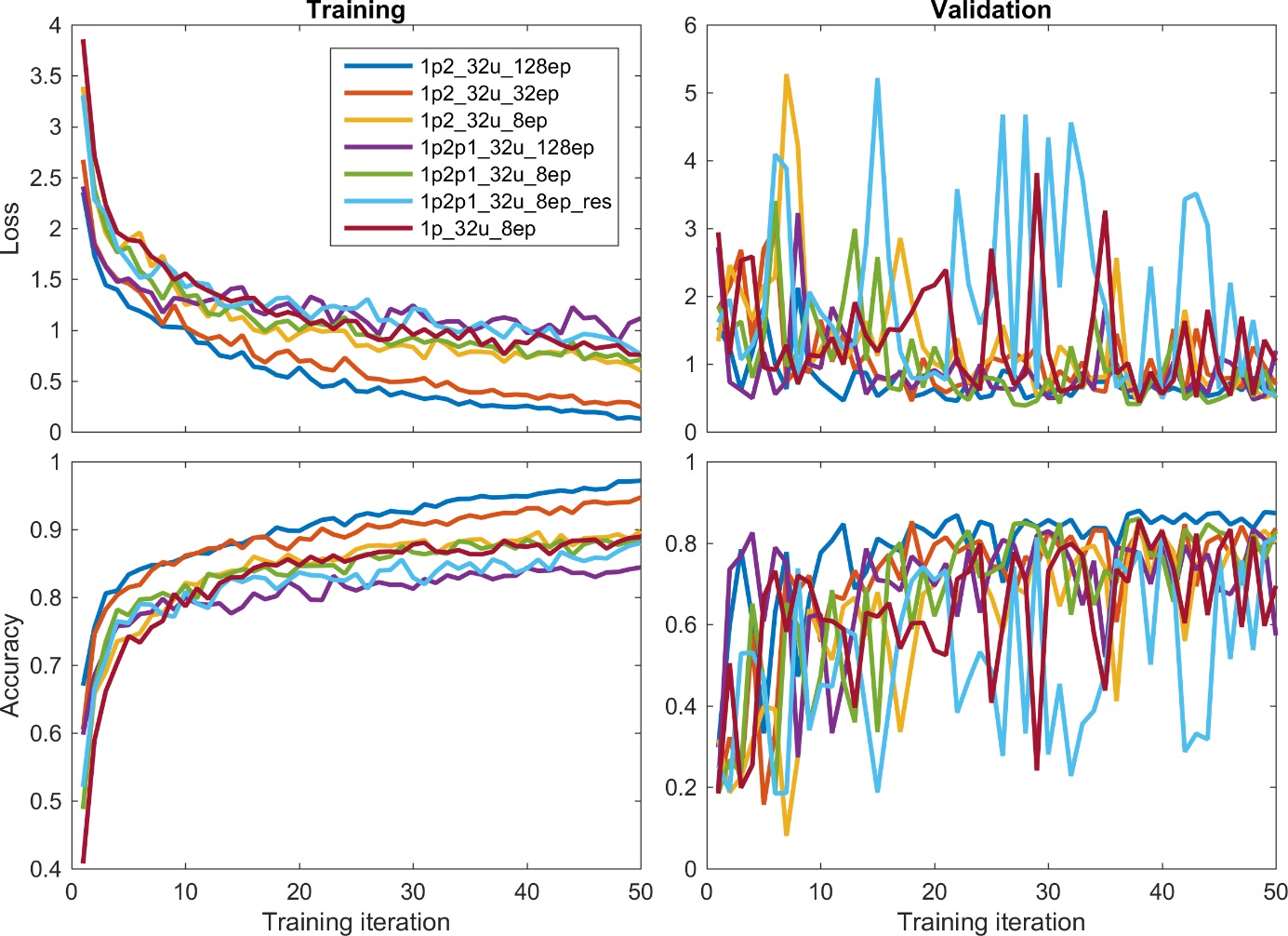


**Figure S5.** Learning curves of CNN_LSTM neuronal networks with raw data as input, trained on dataset 1 (healthy participants). For details see Figure S3.


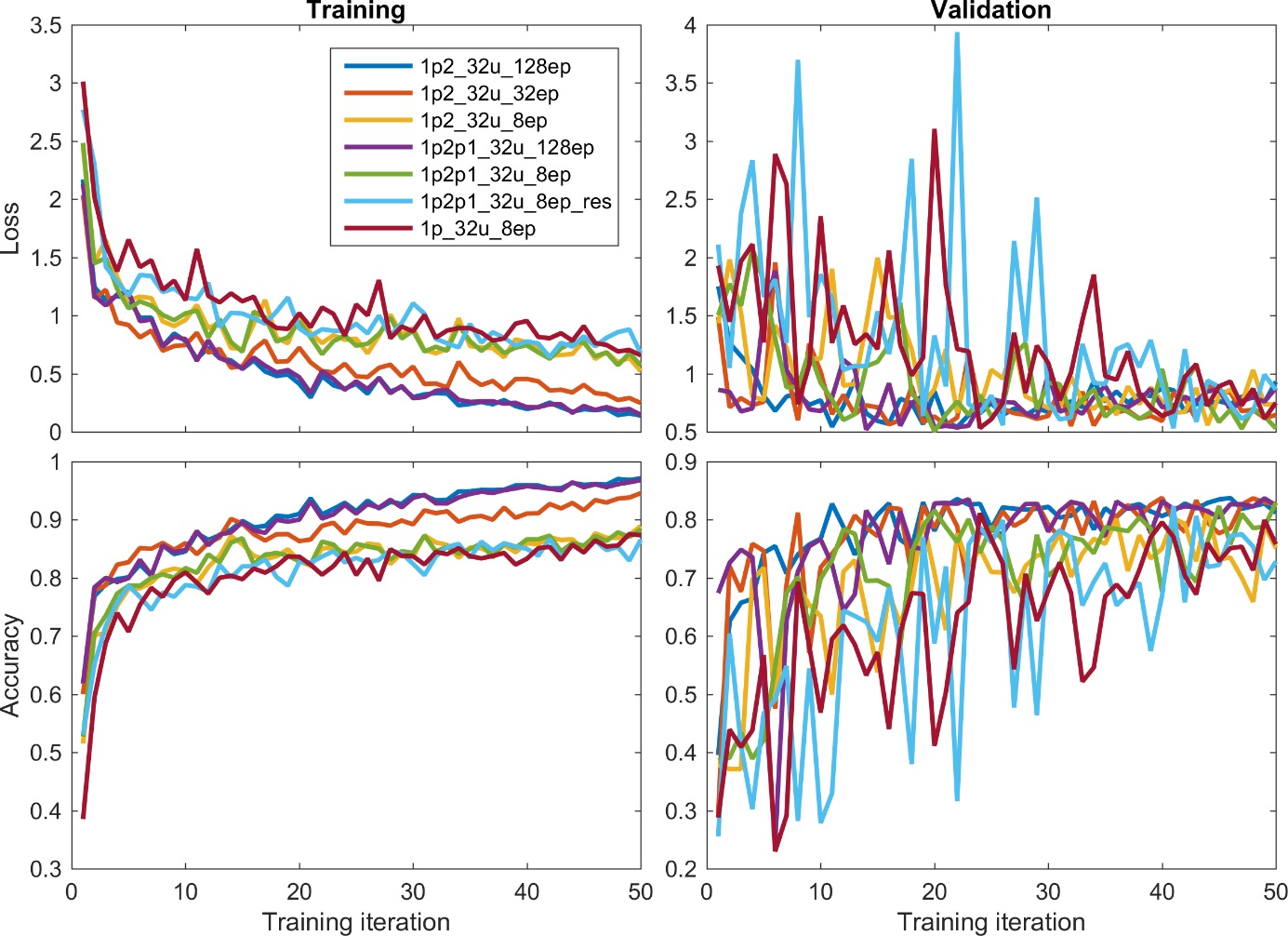


**Figure S6.** Learning curves of CNN_LSTM neuronal networks with raw data as input, trained on a mixture of healthy subjects and patients (datasets 1 and 2). For details see Figure S3.

# Performance evaluation


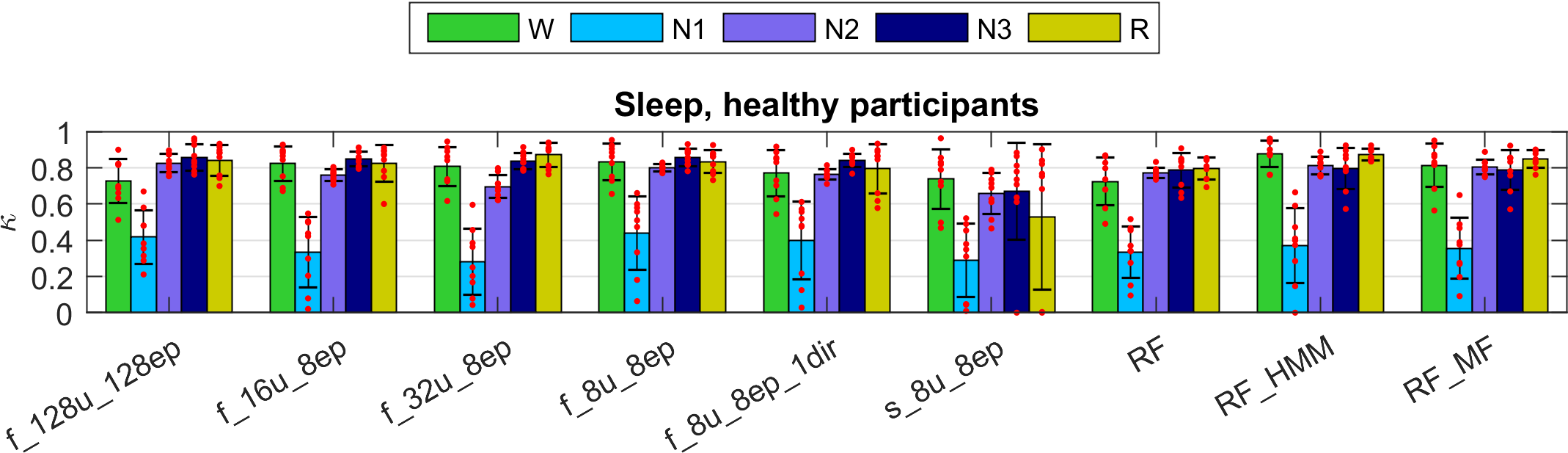


**Figure S7.** Cohen’s kappa obtained with LSTM networks and RF classifiers and features as input. The algorithms were applied to the validation set (9 recordings of dataset 1) of healthy subjects. The first 6 groups of bars represent various neuronal networks. Values for RF classifiers are shown for comparison. See text in supplementary material for the naming conventions of the classifiers. Mean ± SD are shown; red dots represent kappa values of single recordings. Feature vectors were computed for consecutive 20-s epochs and contained 20 features. For the exact performance on the validation and the test set see Table S1.


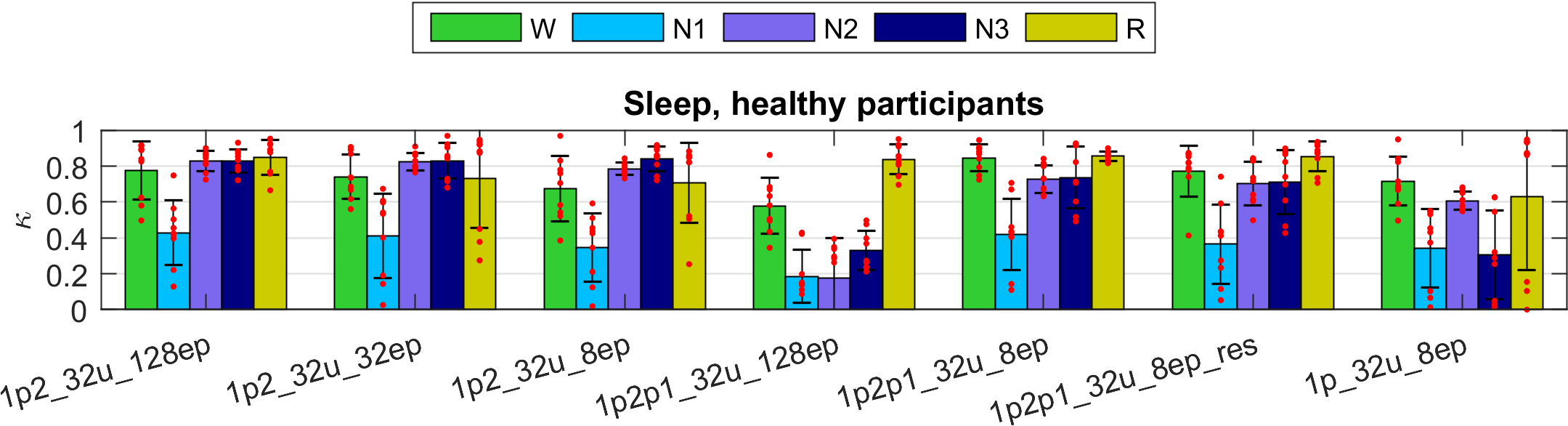


**Figure S8.** Cohen’s kappa obtained with CNN-LSTM networks and raw data as input. The algorithms were applied to the validation set of dataset 1 (healthy subjects). See text in supplementary material for the naming conventions of the classifiers and Figure S7 for further details. For the exact performance on the validation and the test set see Table S2.


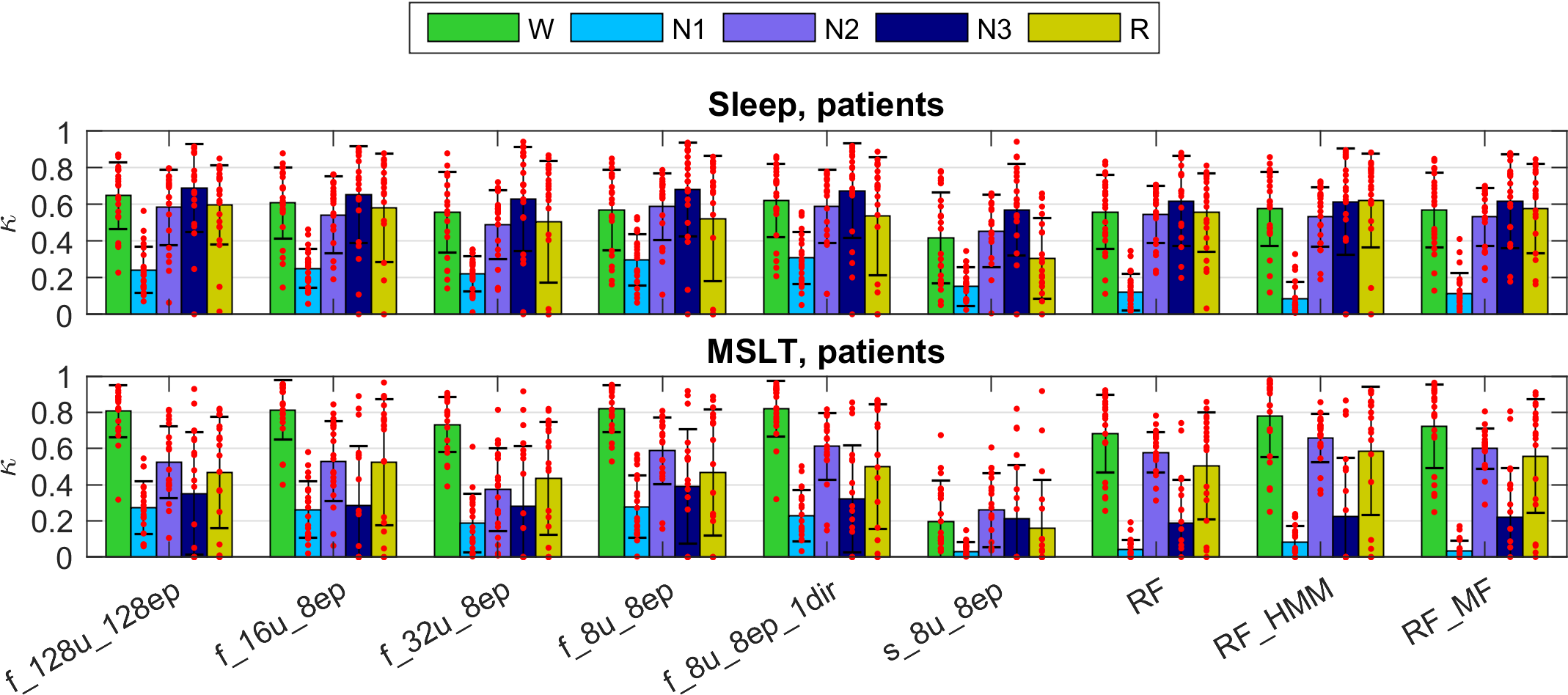


**Figure S9.** Cohen’s kappa obtained with LSTM networks and RF classifiers trained on the healthy subjects applied to patient data (dataset 2). The classifiers were the same as in Figure S7. Top: night sleep recordings; bottom: MSLT recordings. Note that some MSLT recordings did not contain any stage 3 epochs. Such recordings were not taken into account in the computation of the average kappa and the standard deviation for stage 3 (N3). For further details see Figure S7. For the exact performance see Table S3.


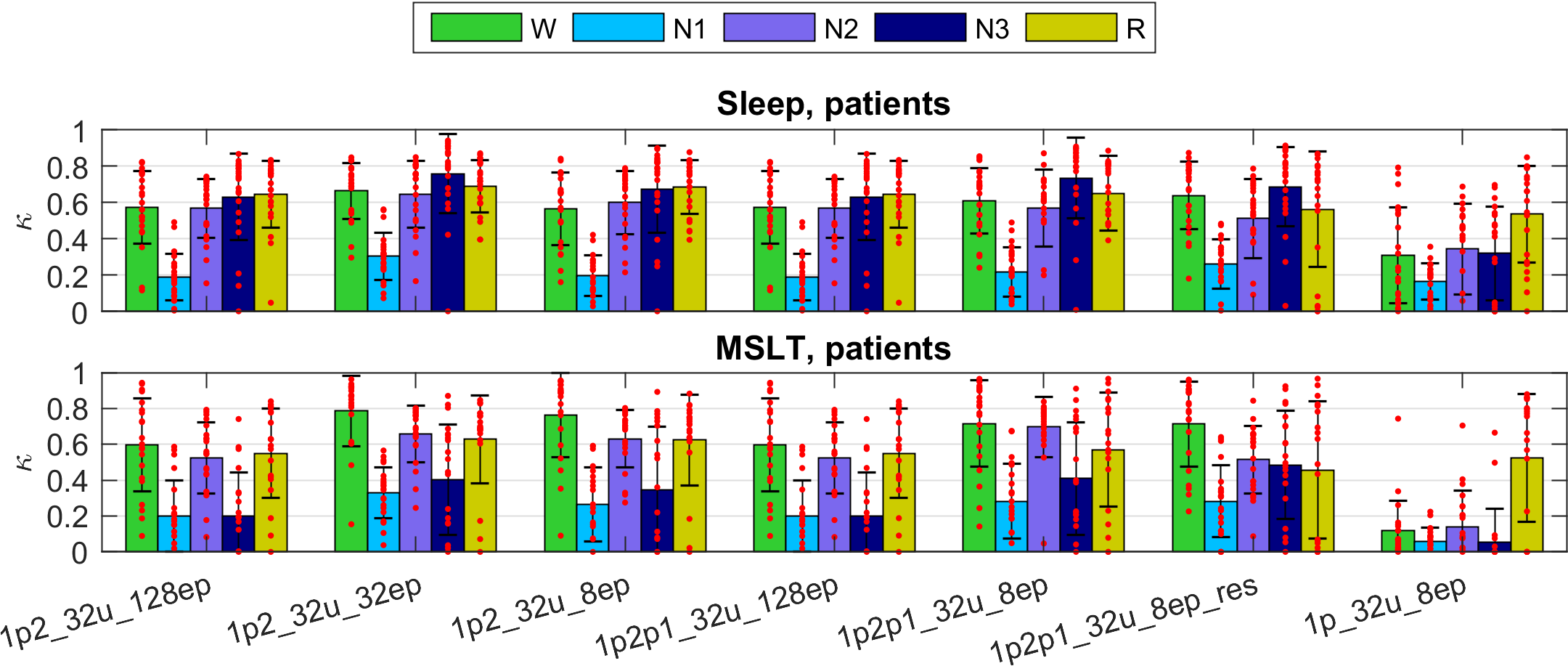


**Figure S10.** Cohen’s kappa obtained with CNN-LSTM networks and raw data as input trained on the healthy subjects and applied to the patient data (dataset 2). Top: sleep recordings; bottom: MSLT recordings. Note that some MSLT recordings did not contain any stage 3 epochs. These recordings were not considered in the computation of the average of kappa and standard deviation for stage 3 (N3). See text in supplementary material for the naming conventions of the classifiers and Suppl. Figure S7 for further details. For the exact performance see Table S4.


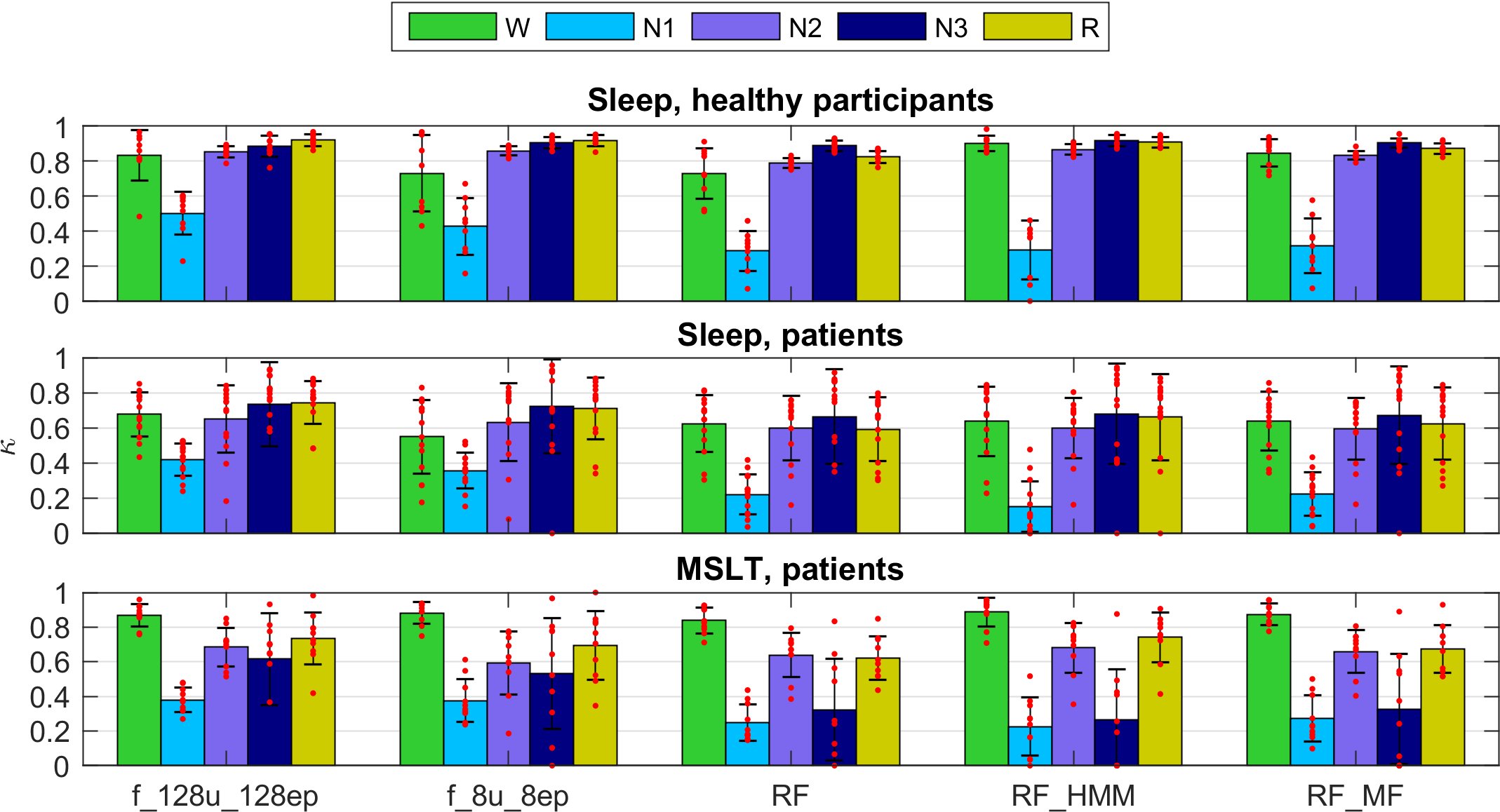


**Figure S11.** Cohen’s kappa obtained with LSTM networks and RF classifiers trained on a mixture of data of patients and healthy participants. See text in supplementary material for the naming conventions of the classifiers. ***Top***: results of the test set (9 recordings) of dataset 1; ***middle***: results of sleep recordings of test data of dataset 2 (patients); ***bottom***: results of MSLT recordings of test data of dataset 2 (patients). For the exact performance see Table S5.


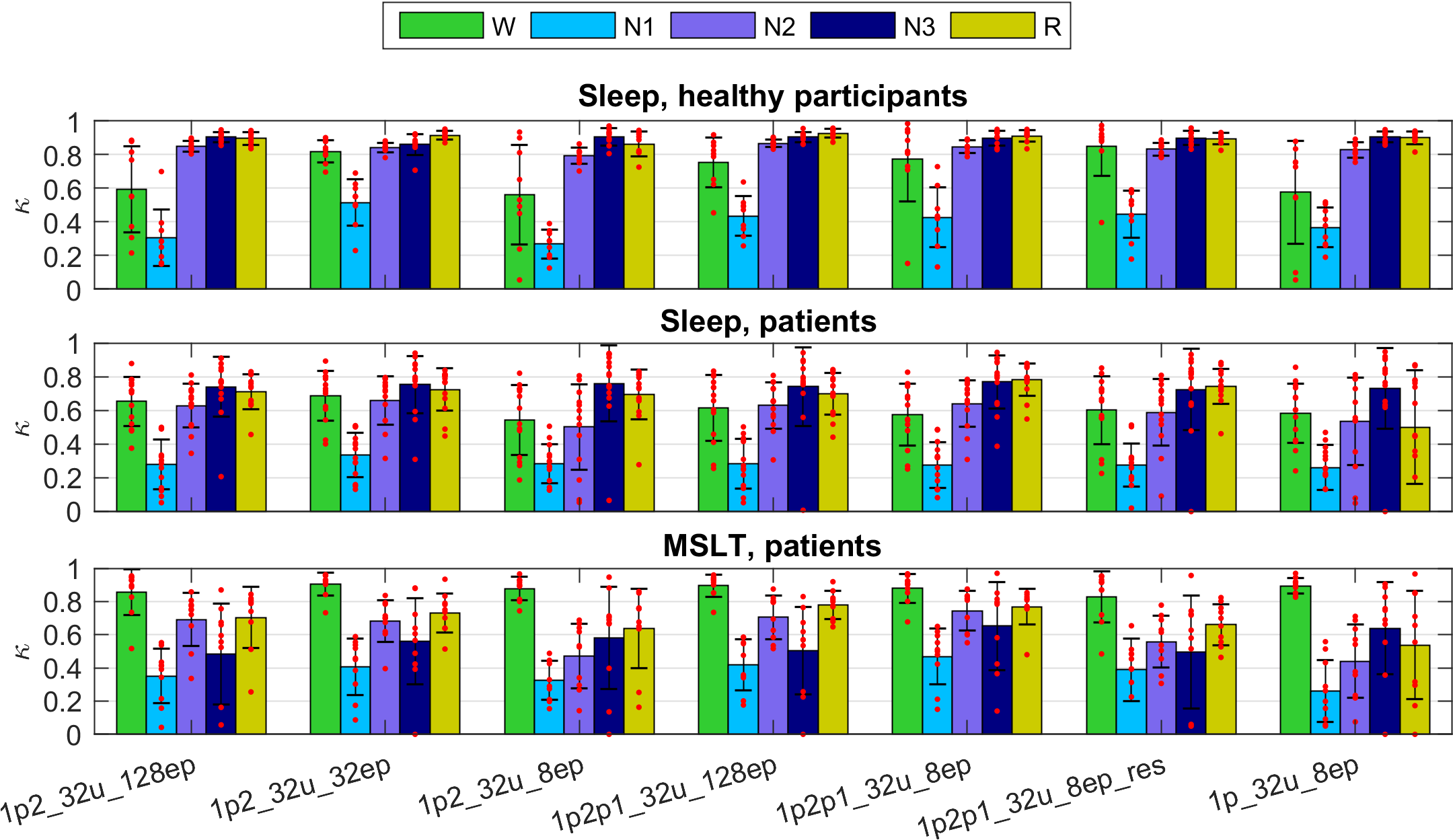


**Figure S12.** Cohen’s kappa obtained with CNN-LSTM networks and raw data as input trained on a mixture of data of patients and healthy participants. ***Top***: results of the test set (9 recordings) of dataset 1; ***middle***: results of sleep recordings of test data of dataset 2 (patients); ***bottom***: results of MSLT recordings of test data of dataset 2 (patients). See text in supplementary material for the naming conventions of the classifiers and Figure S7 for further details. For the exact performance see Table S6.

|  | Validation | | | | |
| --- | --- | --- | --- | --- | --- |
|  | W | 1 | 2 | 3 | R |
| 128f_128u_3l | 0.73 (0.12) | 0.42 (0.15) | 0.83 (0.05) | 0.86 (0.07) | 0.84 (0.08) |
| 8f_16u_3l | 0.82 (0.10) | 0.33 (0.19) | 0.76 (0.03) | 0.85 (0.04) | 0.82 (0.10) |
| 8f_32u_3l | 0.81 (0.11) | 0.28 (0.18) | 0.69 (0.06) | 0.84 (0.04) | 0.87 (0.07) |
| 8f_8u_3l | 0.83 (0.10) | 0.44 (0.20) | 0.80 (0.02) | 0.86 (0.05) | 0.83 (0.06) |
| 8f_8u_3l_1dir | 0.77 (0.13) | 0.40 (0.22) | 0.76 (0.03) | 0.84 (0.04) | 0.79 (0.14) |
| 8s_8u_3l | 0.74 (0.16) | 0.29 (0.20) | 0.66 (0.11) | 0.67 (0.27) | 0.53 (0.40) |
| RF | 0.72 (0.13) | 0.33 (0.14) | 0.77 (0.03) | 0.79 (0.10) | 0.80 (0.06) |
| RFHMM | 0.88 (0.07) | 0.37 (0.21) | 0.81 (0.05) | 0.80 (0.11) | 0.87 (0.03) |
| RFMF | 0.81 (0.12) | 0.36 (0.17) | 0.80 (0.04) | 0.79 (0.11) | 0.85 (0.05) |
|  | Test | | | | |
|  | W | 1 | 2 | 3 | R |
| 128f_128u_3l | 0.86 (0.08) | 0.47 (0.13) | 0.85 (0.04) | 0.90 (0.05) | 0.92 (0.03) |
| 8f_16u_3l | 0.93 (0.01) | 0.47 (0.13) | 0.85 (0.02) | 0.92 (0.03) | 0.92 (0.04) |
| 8f_32u_3l | 0.90 (0.05) | 0.38 (0.14) | 0.84 (0.02) | 0.92 (0.03) | 0.90 (0.04) |
| 8f_8u_3l | 0.92 (0.04) | 0.47 (0.14) | 0.86 (0.02) | 0.93 (0.03) | 0.91 (0.04) |
| 8f_8u_3l_1dir | 0.85 (0.09) | 0.42 (0.10) | 0.84 (0.03) | 0.91 (0.03) | 0.89 (0.04) |
| 8s_8u_3l | 0.76 (0.17) | 0.19 (0.07) | 0.74 (0.06) | 0.82 (0.08) | 0.53 (0.15) |
| RF | 0.73 (0.14) | 0.33 (0.14) | 0.77 (0.03) | 0.79 (0.09) | 0.79 (0.06) |
| RFHMM | 0.91 (0.04) | 0.31 (0.15) | 0.86 (0.03) | 0.91 (0.03) | 0.89 (0.05) |
| RFMF | 0.83 (0.11) | 0.30 (0.16) | 0.83 (0.02) | 0.90 (0.03) | 0.86 (0.06) |

**Table S1.** Cohen’s kappa of the feature-based algorithms on the validation (top part) and test set (bottom part) of dataset 1 (healthy participants). Mean values (standard deviations) are shown. See supplementary material for the naming of the algorithms. W: waking; 1 - 3: NREM sleep stages; R: REM sleep.

|  | Validation | | | | |
| --- | --- | --- | --- | --- | --- |
|  | W | 1 | 2 | 3 | R |
| 1p2_32u_128ep | 0.78 (0.16) | 0.43 (0.18) | 0.83 (0.06) | 0.83 (0.06) | 0.85 (0.10) |
| 1p2_32u_32ep | 0.74 (0.12) | 0.41 (0.24) | 0.82 (0.05) | 0.83 (0.10) | 0.73 (0.28) |
| 1p2_32u_8ep | 0.67 (0.18) | 0.35 (0.19) | 0.78 (0.03) | 0.84 (0.07) | 0.71 (0.22) |
| 1p2p1_32u_128ep | 0.58 (0.15) | 0.18 (0.15) | 0.18 (0.22) | 0.33 (0.11) | 0.84 (0.08) |
| 1p2p1_32u_8ep | 0.84 (0.07) | 0.42 (0.20) | 0.73 (0.08) | 0.74 (0.17) | 0.85 (0.03) |
| 1p2p1_32u_8ep_res | 0.77 (0.14) | 0.37 (0.22) | 0.70 (0.12) | 0.71 (0.18) | 0.85 (0.08) |
| 1p_32u_8ep | 0.72 (0.14) | 0.34 (0.22) | 0.61 (0.05) | 0.31 (0.25) | 0.63 (0.41) |
|  | Test | | | | |
|  | W | 1 | 2 | 3 | R |
| 1p2_32u_128ep | 0.87 (0.08) | 0.52 (0.12) | 0.92 (0.02) | 0.91 (0.03) | 0.94 (0.02) |
| 1p2_32u_32ep | 0.86 (0.11) | 0.51 (0.15) | 0.92 (0.02) | 0.92 (0.04) | 0.95 (0.03) |
| 1p2_32u_8ep | 0.81 (0.24) | 0.50 (0.11) | 0.92 (0.01) | 0.93 (0.02) | 0.94 (0.03) |
| 1p2p1_32u_128ep | 0.65 (0.30) | 0.20 (0.22) | 0.32 (0.17) | 0.53 (0.08) | 0.89 (0.05) |
| 1p2p1_32u_8ep | 0.86 (0.08) | 0.50 (0.19) | 0.89 (0.05) | 0.88 (0.06) | 0.94 (0.03) |
| 1p2p1_32u_8ep_res | 0.89 (0.04) | 0.35 (0.21) | 0.84 (0.09) | 0.84 (0.09) | 0.93 (0.04) |
| 1p_32u_8ep | 0.83 (0.16) | 0.44 (0.10) | 0.84 (0.03) | 0.69 (0.09) | 0.92 (0.03) |

**Table S2.** Cohen’s kappa of the raw data based algorithms on the validation (top part) and test set (bottom part) of dataset 1 (healthy participants). Mean values (standard deviations) are shown. See supplementary material for the naming of the algorithms. W: waking; 1 - 3: NREM sleep stages; R: REM sleep.

|  | PSG | | | | |
| --- | --- | --- | --- | --- | --- |
|  | W | 1 | 2 | 3 | R |
| 128f_128u_3l | 0.65 (0.18) | 0.24 (0.13) | 0.58 (0.21) | 0.69 (0.24) | 0.60 (0.22) |
| 8f_16u_3l | 0.61 (0.19) | 0.25 (0.11) | 0.54 (0.21) | 0.65 (0.26) | 0.58 (0.30) |
| 8f_32u_3l | 0.56 (0.22) | 0.22 (0.10) | 0.49 (0.19) | 0.63 (0.28) | 0.50 (0.33) |
| 8f_8u_3l | 0.57 (0.22) | 0.30 (0.14) | 0.59 (0.18) | 0.68 (0.26) | 0.52 (0.34) |
| 8f_8u_3l_1dir | 0.62 (0.20) | 0.31 (0.14) | 0.59 (0.20) | 0.67 (0.26) | 0.54 (0.32) |
| 8s_8u_3l | 0.42 (0.25) | 0.15 (0.11) | 0.45 (0.20) | 0.57 (0.25) | 0.30 (0.22) |
| RF | 0.56 (0.20) | 0.12 (0.10) | 0.55 (0.16) | 0.62 (0.25) | 0.55 (0.21) |
| RFHMM | 0.58 (0.20) | 0.08 (0.09) | 0.53 (0.16) | 0.61 (0.29) | 0.62 (0.26) |
| RFMF | 0.57 (0.20) | 0.11 (0.11) | 0.53 (0.16) | 0.62 (0.26) | 0.58 (0.24) |
|  | MSLT | | | | |
|  | W | 1 | 2 | 3 | R |
| 128f_128u_3l | 0.81 (0.14) | 0.27 (0.15) | 0.52 (0.20) | 0.35 (0.34) | 0.47 (0.31) |
| 8f_16u_3l | 0.81 (0.16) | 0.26 (0.16) | 0.53 (0.22) | 0.29 (0.33) | 0.52 (0.35) |
| 8f_32u_3l | 0.73 (0.15) | 0.19 (0.16) | 0.37 (0.23) | 0.28 (0.34) | 0.43 (0.31) |
| 8f_8u_3l | 0.82 (0.13) | 0.28 (0.17) | 0.59 (0.18) | 0.39 (0.32) | 0.47 (0.35) |
| 8f_8u_3l_1dir | 0.82 (0.15) | 0.23 (0.14) | 0.61 (0.18) | 0.32 (0.30) | 0.50 (0.34) |
| 8s_8u_3l | 0.19 (0.23) | 0.03 (0.05) | 0.26 (0.20) | 0.21 (0.30) | 0.16 (0.27) |
| RF | 0.68 (0.21) | 0.04 (0.06) | 0.58 (0.11) | 0.19 (0.24) | 0.50 (0.29) |
| RFHMM | 0.78 (0.23) | 0.08 (0.09) | 0.66 (0.13) | 0.23 (0.32) | 0.59 (0.35) |
| RFMF | 0.72 (0.23) | 0.03 (0.06) | 0.60 (0.11) | 0.22 (0.27) | 0.56 (0.31) |

**Table S3.** Cohen’s kappa values of the feature-based algorithms trained on dataset 1 (healthy participants), i.e. the same models as in the Table S1, but validated on dataset 2 (patients). Sleep recordings (top part) and MSLT recordings (bottom part) were analyzed separately. Mean values (standard deviations) are shown. See supplementary material for the naming of the algorithms. W: waking; 1 - 3: NREM sleep stages; R: REM sleep.

|  | PSG | | | | |
| --- | --- | --- | --- | --- | --- |
|  | W | 1 | 2 | 3 | R |
| 1p2_32u_128ep | 0.57 (0.20) | 0.19 (0.13) | 0.57 (0.16) | 0.63 (0.24) | 0.64 (0.18) |
| 1p2_32u_32ep | 0.66 (0.15) | 0.30 (0.13) | 0.65 (0.19) | 0.76 (0.22) | 0.69 (0.15) |
| 1p2_32u_8ep | 0.57 (0.20) | 0.20 (0.11) | 0.60 (0.17) | 0.67 (0.24) | 0.68 (0.15) |
| 1p2p1_32u_128ep | 0.57 (0.20) | 0.19 (0.13) | 0.57 (0.16) | 0.63 (0.24) | 0.64 (0.18) |
| 1p2p1_32u_8ep | 0.61 (0.18) | 0.22 (0.14) | 0.57 (0.21) | 0.73 (0.22) | 0.65 (0.21) |
| 1p2p1_32u_8ep_res | 0.64 (0.19) | 0.26 (0.14) | 0.51 (0.22) | 0.69 (0.22) | 0.56 (0.32) |
| 1p_32u_8ep | 0.31 (0.26) | 0.16 (0.10) | 0.34 (0.25) | 0.32 (0.26) | 0.54 (0.27) |
|  | MSLT | | | | |
|  | W | 1 | 2 | 3 | R |
| 1p2_32u_128ep | 0.60 (0.26) | 0.20 (0.20) | 0.52 (0.20) | 0.19 (0.24) | 0.52 (0.27) |
| 1p2_32u_32ep | 0.79 (0.19) | 0.33 (0.14) | 0.66 (0.16) | 0.40 (0.31) | 0.60 (0.28) |
| 1p2_32u_8ep | 0.76 (0.23) | 0.26 (0.21) | 0.63 (0.16) | 0.33 (0.35) | 0.59 (0.28) |
| 1p2p1_32u_128ep | 0.60 (0.26) | 0.20 (0.20) | 0.52 (0.20) | 0.19 (0.24) | 0.52 (0.27) |
| 1p2p1_32u_8ep | 0.72 (0.24) | 0.28 (0.21) | 0.70 (0.17) | 0.41 (0.31) | 0.54 (0.33) |
| 1p2p1_32u_8ep_res | 0.71 (0.24) | 0.28 (0.20) | 0.52 (0.19) | 0.49 (0.30) | 0.43 (0.39) |
| 1p_32u_8ep | 0.12 (0.17) | 0.06 (0.08) | 0.14 (0.20) | 0.05 (0.18) | 0.47 (0.37) |

**Table S4.** Cohen’s kappa values of the raw-data based algorithms, trained on dataset 1 (healthy participants), i.e. the same models as in the Table S2, but they were validated on dataset 2 (patients). Sleep recordings (top part) and MSLT recordings (bottom part) were analyzed separately. Mean values (standard deviations) are shown. See supplementary material for the naming of the algorithms. W: waking; 1 - 3: NREM sleep stages; R: REM sleep.

|  | Sleep healthy participants | | | | |
| --- | --- | --- | --- | --- | --- |
|  | W | 1 | 2 | 3 | R |
| 128f_128u_3l_dp25 | 0.83 (0.14) | 0.50 (0.12) | 0.85 (0.03) | 0.88 (0.06) | 0.92 (0.03) |
| 8f_8u_3l_dp25 | 0.73 (0.22) | 0.43 (0.16) | 0.86 (0.03) | 0.90 (0.03) | 0.92 (0.03) |
| RF | 0.73 (0.15) | 0.29 (0.11) | 0.79 (0.03) | 0.89 (0.03) | 0.82 (0.03) |
| RFHMM | 0.90 (0.04) | 0.29 (0.17) | 0.87 (0.03) | 0.92 (0.03) | 0.91 (0.03) |
| RFMF | 0.85 (0.08) | 0.32 (0.16) | 0.83 (0.02) | 0.90 (0.03) | 0.87 (0.03) |
|  | Sleep patients | | | | |
|  | W | 1 | 2 | 3 | R |
| 128f_128u_3l_dp25 | 0.68 (0.13) | 0.42 (0.09) | 0.65 (0.19) | 0.74 (0.24) | 0.75 (0.12) |
| 8f_8u_3l_dp25 | 0.55 (0.21) | 0.36 (0.10) | 0.63 (0.22) | 0.72 (0.27) | 0.71 (0.18) |
| RF | 0.63 (0.16) | 0.22 (0.11) | 0.60 (0.18) | 0.67 (0.27) | 0.59 (0.18) |
| RFHMM | 0.64 (0.20) | 0.15 (0.14) | 0.60 (0.17) | 0.68 (0.29) | 0.66 (0.25) |
| RFMF | 0.64 (0.17) | 0.22 (0.12) | 0.60 (0.18) | 0.67 (0.28) | 0.63 (0.21) |
|  | MSLT patients | | | | |
|  | W | 1 | 2 | 3 | R |
| 128f_128u_3l_dp25 | 0.87 (0.06) | 0.38 (0.07) | 0.68 (0.11) | 0.62 (0.27) | 0.74 (0.15) |
| 8f_8u_3l_dp25 | 0.88 (0.06) | 0.38 (0.12) | 0.59 (0.18) | 0.53 (0.32) | 0.69 (0.20) |
| RF | 0.84 (0.07) | 0.25 (0.11) | 0.64 (0.13) | 0.32 (0.29) | 0.62 (0.13) |
| RFHMM | 0.89 (0.08) | 0.23 (0.17) | 0.68 (0.14) | 0.27 (0.29) | 0.74 (0.14) |
| RFMF | 0.87 (0.06) | 0.27 (0.13) | 0.66 (0.12) | 0.33 (0.32) | 0.67 (0.14) |

**Table S5.** Cohen’s kappa values of the feature-based algorithms trained on both datasets (healthy participants and patients). They were validated on the corresponding test parts. Validation and test parts of dataset 1 were merged (18 recordings). The table divided into three parts: sleep recordings of healthy participants (top part), sleep recordings of patients (middle part) and MSLT recordings (bottom part). Mean values (standard deviations) are shown. See supplementary material for the naming of the algorithms. W: waking; 1 - 3: NREM sleep stages; R: REM sleep.

|  | Sleep healthy participants | | | | |
| --- | --- | --- | --- | --- | --- |
|  | W | 1 | 2 | 3 | R |
| 1p2_32u_128ep | 0.59 (0.26) | 0.30 (0.17) | 0.85 (0.03) | 0.90 (0.03) | 0.89 (0.04) |
| 1p2_32u_32ep | 0.82 (0.07) | 0.51 (0.14) | 0.84 (0.03) | 0.86 (0.06) | 0.91 (0.02) |
| 1p2_32u_8ep | 0.56 (0.30) | 0.27 (0.08) | 0.79 (0.05) | 0.90 (0.05) | 0.86 (0.07) |
| 1p2p1_32u_128ep | 0.75 (0.15) | 0.43 (0.12) | 0.87 (0.02) | 0.90 (0.03) | 0.93 (0.03) |
| 1p2p1_32u_8ep | 0.77 (0.25) | 0.42 (0.18) | 0.84 (0.04) | 0.90 (0.04) | 0.91 (0.04) |
| 1p2p1_32u_8ep_res | 0.85 (0.18) | 0.44 (0.14) | 0.83 (0.04) | 0.90 (0.04) | 0.89 (0.03) |
| 1p_32u_8ep | 0.57 (0.31) | 0.37 (0.12) | 0.83 (0.04) | 0.90 (0.03) | 0.90 (0.04) |
| RF | 0.74 (0.15) | 0.30 (0.13) | 0.88 (0.02) | 0.91 (0.02) | 0.88 (0.03) |
| RFHMM | 0.91 (0.04) | 0.30 (0.17) | 0.92 (0.02) | 0.93 (0.03) | 0.93 (0.03) |
| RFMF | 0.86 (0.07) | 0.33 (0.16) | 0.90 (0.02) | 0.92 (0.02) | 0.91 (0.02) |
|  | Sleep patients | | | | |
|  | W | 1 | 2 | 3 | R |
| 1p2_32u_128ep | 0.65 (0.15) | 0.28 (0.15) | 0.63 (0.13) | 0.74 (0.18) | 0.71 (0.10) |
| 1p2_32u_32ep | 0.69 (0.15) | 0.34 (0.13) | 0.66 (0.15) | 0.75 (0.17) | 0.72 (0.13) |
| 1p2_32u_8ep | 0.54 (0.21) | 0.28 (0.12) | 0.50 (0.25) | 0.76 (0.23) | 0.70 (0.15) |
| 1p2p1_32u_128ep | 0.62 (0.20) | 0.28 (0.15) | 0.63 (0.14) | 0.74 (0.23) | 0.70 (0.12) |
| 1p2p1_32u_8ep | 0.58 (0.18) | 0.28 (0.13) | 0.64 (0.14) | 0.77 (0.16) | 0.78 (0.10) |
| 1p2p1_32u_8ep_res | 0.60 (0.20) | 0.28 (0.13) | 0.59 (0.20) | 0.73 (0.24) | 0.74 (0.10) |
| 1p_32u_8ep | 0.58 (0.18) | 0.26 (0.13) | 0.54 (0.26) | 0.73 (0.24) | 0.50 (0.34) |
| RF | 0.68 (0.12) | 0.30 (0.15) | 0.75 (0.17) | 0.67 (0.27) | 0.66 (0.18) |
| RFHMM | 0.69 (0.14) | 0.19 (0.16) | 0.75 (0.16) | 0.67 (0.29) | 0.72 (0.24) |
| RFMF | 0.69 (0.13) | 0.30 (0.16) | 0.75 (0.16) | 0.69 (0.28) | 0.67 (0.20) |
|  | MSLT patients | | | | |
|  | W | 1 | 2 | 3 | R |
| 1p2_32u_128ep | 0.86 (0.14) | 0.35 (0.16) | 0.69 (0.16) | 0.49 (0.30) | 0.70 (0.19) |
| 1p2_32u_32ep | 0.90 (0.07) | 0.41 (0.17) | 0.68 (0.12) | 0.56 (0.26) | 0.73 (0.12) |
| 1p2_32u_8ep | 0.88 (0.07) | 0.33 (0.12) | 0.47 (0.19) | 0.58 (0.31) | 0.64 (0.24) |
| 1p2p1_32u_128ep | 0.90 (0.07) | 0.42 (0.15) | 0.70 (0.13) | 0.50 (0.26) | 0.78 (0.08) |
| 1p2p1_32u_8ep | 0.88 (0.09) | 0.47 (0.17) | 0.74 (0.12) | 0.65 (0.26) | 0.77 (0.11) |
| 1p2p1_32u_8ep_res | 0.83 (0.15) | 0.39 (0.19) | 0.56 (0.16) | 0.50 (0.34) | 0.66 (0.12) |
| 1p_32u_8ep | 0.89 (0.05) | 0.26 (0.19) | 0.44 (0.22) | 0.64 (0.28) | 0.54 (0.33) |
| RF | 0.95 (0.02) | 0.28 (0.11) | 0.68 (0.12) | 0.32 (0.28) | 0.65 (0.12) |
| RFHMM | 0.97 (0.03) | 0.25 (0.17) | 0.72 (0.13) | 0.18 (0.30) | 0.76 (0.16) |
| RFMF | 0.96 (0.02) | 0.29 (0.13) | 0.69 (0.12) | 0.31 (0.30) | 0.70 (0.13) |

**Table S6**. Cohen’s kappa values of the raw-data based algorithms trained on both datasets (healthy participants and patients). They were validated on the corresponding test parts. Validation and test parts of dataset 1 were merged (18 recordings). The table divided into three parts: sleep recordings of healthy participants (top part), sleep recordings of patients (middle part) and MSLT recordings (bottom part). Mean values (standard deviations) are shown. See supplementary material for the naming of the algorithms. W: waking; 1 - 3: NREM sleep stages; R: REM sleep.

# Supplementary references

Aeschbach, D., and Borbély, A. (1993). All‐night dynamics of the human sleep EEG. *Journal of sleep research* 2**,** 70-81.

Berger, H. (1929). Über das elektrenkephalogramm des menschen. *European Archives of Psychiatry and Clinical Neuroscience* 87**,** 527-570.

Bersagliere, A., and Achermann, P. (2010). Slow oscillations in human non‐rapid eye movement sleep electroencephalogram: effects of increased sleep pressure. *Journal of sleep research* 19**,** 228-237.

Bishop, C. (2016). *Pattern recognition and machine learning.* Springer-Verlag New York.

Borbély, A.A., Baumann, F., Brandeis, D., Strauch, I., and Lehmann, D. (1981). Sleep deprivation: effect on sleep stages and EEG power density in man. *Electroencephalography and clinical neurophysiology* 51**,** 483-493.

Carl, C., Açık, A., König, P., Engel, A.K., and Hipp, J.F. (2012). The saccadic spike artifact in MEG. *Neuroimage* 59**,** 1657-1667.

Drummond, J., Brann, C., Perkins, D., and Wolfe, D. (1991). A comparison of median frequency, spectral edge frequency, a frequency band power ratio, total power, and dominance shift in the determination of depth of anesthesia. *Acta Anaesthesiologica Scandinavica* 35**,** 693-699.

Du Bois-Reymond, E. (1848). *Untersuchungen über thierische Elektricität.* G. Reimer.

Hinton, G.E., Srivastava, N., Krizhevsky, A., Sutskever, I., and Salakhutdinov, R.R. (2012). Improving neural networks by preventing co-adaptation of feature detectors. *arXiv preprint arXiv:1207.0580*.

Imtiaz, S.A., and Rodriguez-Villegas, E. (2014). A low computational cost algorithm for REM sleep detection using single channel EEG. *Annals of biomedical engineering* 42**,** 2344-2359.

Iwasaki, M., Kellinghaus, C., Alexopoulos, A.V., Burgess, R.C., Kumar, A.N., Han, Y.H., Lüders, H.O., and Leigh, R.J. (2005). Effects of eyelid closure, blinks, and eye movements on the electroencephalogram. *Clinical Neurophysiology* 116**,** 878-885.

Kingma, D., and Ba, J. (2014). Adam: A method for stochastic optimization. *arXiv preprint arXiv:1412.6980*.

Lessard, C., and Paschall, R. (1970). A system for quantifying EEG slow wave activity. *Electroencephalography and clinical neurophysiology* 29**,** 516-520.

Louis, R.P., Lee, J., and Stephenson, R. (2004). Design and validation of a computer-based sleep-scoring algorithm. *Journal of neuroscience methods* 133**,** 71-80.

Magosso, E., Provini, F., Montagna, P., and Ursino, M. (2006). A wavelet based method for automatic detection of slow eye movements: A pilot study. *Medical engineering & physics* 28**,** 860-875.

Nesterov, Y. (Year). "A method of solving a convex programming problem with convergence rate O (1/k2)", in: *Soviet Mathematics Doklady*), 372-376.

Ogilvie, R.D., Mcdonagh, D.M., Stone, S.N., and Wilkinson, R.T. (1988). Eye movements and the detection of sleep onset. *Psychophysiology* 25**,** 81-91.

Pop-Jordanova, N., and Pop-Jordanov, J. (2005). Spectrum-weighted EEG frequency (“brain-rate”) as a quantitative indicator of mental arousal. *Prilozi* 26**,** 35-42.

Rechtschaffen, A., and Kales, A. (1968). *A manual of standardized terminology, techniques and scoring system for sleep stages of human subjects.* Bethseda, Maryland: National Institutes of Health.

Schlögl, A., and Brunner, C. (2008). BioSig: A Free and Open Source Software Library for BCI Research. *Computer* 41**,** 44-50.

Srivastava, N., Hinton, G.E., Krizhevsky, A., Sutskever, I., and Salakhutdinov, R. (2014). Dropout: a simple way to prevent neural networks from overfitting. *Journal of Machine Learning Research* 15**,** 1929-1958.

Sutskever, I., Martens, J., Dahl, G., and Hinton, G. (Year). "On the importance of initialization and momentum in deep learning", in: *International conference on machine learning*), 1139-1147.

Viterbi, A. (1967). Error bounds for convolutional codes and an asymptotically optimum decoding algorithm. *IEEE transactions on Information Theory* 13**,** 260-269.

Werbos, P. (1974). Beyond regression: New tools for prediction and analysis in the behavior science. *Unpublished Doctoral Dissertation, Harvard University*.

Werbos, P.J. (1994). *The roots of backpropagation: from ordered derivatives to neural networks and political forecasting.* John Wiley & Sons.

Young, L.R., and Sheena, D. (1975). Eye-movement measurement techniques. *American Psychologist* 30**,** 315.
